# Bhlhe40 limits early IL-10 production from CD4^+^ T cells during *Plasmodium yoelii* 17X infection

**DOI:** 10.1128/iai.00367-23

**Published:** 2023-10-16

**Authors:** Kara A. O'Neal, Sheldon L. Zeltner, Camille L. Foscue, Jason S. Stumhofer

**Affiliations:** 1 Department of Microbiology and Immunology, University of Arkansas for Medical Sciences, Little Rock, Arkansas, USA; University of California, Davis, California, USA

**Keywords:** *Plasmodium*, cytokines, T cells, infectious disease, transcription factors

## Abstract

The cytokine IL-10 suppresses T-cell-mediated immunity, which is required to control infection with *Plasmodium yoelii*. Consequently, IL-10 can delay the time needed to resolve this infection, leading to a higher parasite burden. While the pathways that lead to IL-10 production by CD4^+^ T cells are well defined, much less is known about the mediators that suppress the expression of this potent anti-inflammatory cytokine. Here, we show that the transcription factor basic helix-loop-helix family member e40 (Bhlhe40) contributes to controlling parasite burden in response to *P. yoelii* infection in mice. Loss of Bhlhe40 expression in mice results in higher *Il10* expression, higher peak parasitemia, and a delay in parasite clearance. The observed phenotype was not due to defects in T-cell activation and proliferation or the humoral response. Nor was it due to changes in regulatory T-cell numbers. However, blocking IL-10 signaling reversed the outcome in *Bhlhe40^−/^* ^−^ mice, suggesting that excess IL-10 production limits their ability to control the infection properly. In addition to suppressing *Il10* expression in CD4^+^ T cells, Bhlhe40 can promote *Ifng* expression. Indeed, IFN-γ production by CD4^+^ T cells isolated from the liver was significantly affected by the loss of Bhlhe40. Lastly, Bhlhe40 deletion in T cells resulted in a phenotype similar to that observed in the *Bhlhe40^−/^* ^−^ mice, indicating that Bhlhe40 expression in T cells contributes to the ability of mice to control infection with *P. yoelii*.

## INTRODUCTION

IL-10 is an anti-inflammatory cytokine vital in determining the outcome of infection with *Plasmodium* in mice and humans (1 – 6). During *Plasmodium* infection, IL-10 is produced by many cell types, including B cells, CD4^+^ T cells, DCs, macrophages, NK cells, and γδ T cells (7). Specifically, IL-10 derived from Foxp3^−^CD4^+^ T cells is a critical source of this cytokine during infection (8, 9). IL-10 acts to suppress the activity of T helper-1 (Th1) cells and T follicular helper (Tfh) cells, which are two effector T-cell populations critical for parasite control (7, 10). While IL-10 primarily protects the host against immune-mediated pathology by preventing excessive inflammation, it also restricts Th1 and Tfh cell responses, increasing the time needed to clear the infection and resulting in a higher parasite burden (11). Alternatively, B-cell intrinsic IL-10 signaling is critical for promoting the germinal center (GC) response during *P. yoelii* infection by limiting IFN-γ–driven T-bet expression in B cells, which restricts the humoral response (12, 13). During *P. falciparum* infection in humans, high quantities of IL-10 are associated with an inability to clear the parasite, while reduced amounts of IL-10 contribute to severe anemia (4, 14). Given the crucial role of IL-10 in regulating the immune response to *Plasmodium*, understanding the factors and mechanisms that control its expression offers opportunities to manipulate the production of IL-10 to enhance vaccine or natural immunity.

Recent studies have demonstrated that the transcription factor basic helix-loop-helix family member e40 (Bhlhe40) regulates cytokine production by CD4^+^ T cells in mice and humans (15, 16). Bhlhe40 belongs to a family of basic helix-loop-helix transcriptional regulators that bind E-box DNA motifs (CACGTG) and are expressed in many cell types. This family of transcription factors plays functional roles in regulating apoptosis, cell cycle, and differentiation (15). Depending on the context, the deletion of Bhlhe40 in mice results in either a protective or pathogenic outcome (15). In autoimmune disorders, including colitis, experimental autoimmune encephalitis, and graft-versus-host disease, Bhlhe40 promotes pathology in mice (15, 17). However, in response to infection with intracellular pathogens, Bhlhe40 plays a protective role (18, 19). Specifically, Bhlhe40 expression in CD4^+^ T cells was shown to act as a negative regulator of IL-10 expression during *Mycobacterium tuberculosis* and *Toxoplasma gondii* infection (18, 19). Studies in these infection models showed that when Bhlhe40 is absent in CD4^+^ T cells, IL-10 production increases, while IFN-γ production decreases. Moreover, the decrease in IFN-γ production is partly IL-10–independent, as blocking IL-10R signaling only partially restores IFN-γ production by CD4^+^ T cells *in vitro* and *in vivo*, suggesting regulation of IFN-γ expression by Bhlhe40 (18, 19).

Given the role of Bhlhe40 in regulating IL-10 and IFN-γ expression in other Th1-centric infections, it was hypothesized that *Bhlhe40^−/^* ^−^ mice would have an impaired ability to control infection with *P. yoelii* 17X, a non-lethal strain of the parasite, due to increased production of IL-10 and reduced production of IFN-γ. Findings here demonstrate that Bhlhe40 plays an essential role in controlling infection with *P. yoelii* in mice by repressing IL-10 expression in T cells. Furthermore, the blockade of IL-10R signaling led to a significant reduction in parasite burden in *Bhlhe40^−/^* ^−^ mice, indicating that Bhlhe40 negatively regulates IL-10 production during *P. yoelii* infection resulting in parasite control. While the loss of Bhlhe40 did not significantly impact IFN-γ production by CD4^+^ T cells in the spleen, IFN-γ was significantly reduced in the liver after *P. yoelii* infection.

## MATERIALS AND METHODS

### Mice and infections

In compliance with institutional guidelines, mice were bred and housed in specific-pathogen-free facilities at the University of Arkansas for Medical Sciences (UAMS). The Institutional Animal Care and Use Committee at UAMS approved all procedures on mice in these studies. Male and female mice were used in these studies to account for sex differences in the immune response. The following mouse strains were from The Jackson Laboratory: C57BL/6J, B6.SJL-*Ptprc^a^ Pepc^b^/BoyJ* (CD45.1), *Bhlhe40^−/−^*, *Il10^−/^* ^−^, *Cd4-cre*, and B6.PL-*Thy1^a^*/CyJ. The *Bhlhe40^fl/fl^* mice were a gift from Dr. Edelson (Washington University), and the PbT-II mice were a gift from Dr. Heath (University of Melbourne) (18, 20). The *Bhlhe40^fl/fl^-Cd4-cre* and *Bhlhe40^−/^* ^−^ PbT-II Tg mice were generated in-house.

For *Plasmodium yoelii* 17X (MR4 17X, BEI Resources Repository) infection, cryopreserved parasitized red blood cell (pRBC) stocks were first passaged in a C57BL/6J mouse by injecting thawed stocks intraperitoneally (i.p.). Blood was then collected at the time of sacrifice for infecting experimental mice. Next, 10^5^ pRBCs from the mouse containing the passaged stock were injected i.p. for primary infection of the experimental mice. All experimental mice were used at 7 to 12 weeks of age. Flow cytometry was used to determine blood parasitemia based on a previously described method for all primary infections (21). All procedures involving *P. yoelii* 17X were approved by the Institutional Biosafety Committee at UAMS.

### Real-time quantitative PCR

RNA from splenocytes of naïve and day 5 and 7 infected mice or CD45^+^ leukocytes from the liver of day 7 infected mice were isolated using an RNAeasy kit (Qiagen) followed by DNAse treatment. CD4^+^ T cells were enriched from the spleens of mice infected with *P. yoelii* 17X at 5 days post-infection (p.i.) or livers at day 7 p.i., using anti-CD4 microbeads (Miltenyi Biotec) and an AutoMACS Pro cell separator (Miltenyi Biotech). Enriched T cells were stained with CD4, CD44, and CD11a to identify antigen-experienced CD4^+^ T cells and sorted using a BD FACSAriaIII. Cells were washed with sterile phosphate buffered saline (PBS) and lysed in Trizol (Thermo Fisher). RNA was isolated, and DNAse, treated using a DirectZol RNA MicroPrep kit per the manufacturer’s instructions (Zymo). Superscript III (Invitrogen) was used to prepare complementary DNA. SYBR Green PCR Master Mix (BioRad) and a QuantStudio 6 Flex real-time PCR system (Life Technologies) were used to perform real-time quantitative PCR (RT-qPCR). Primers are listed in Table S1. Data were normalized to the *Hprt* housekeeping gene, and the 2^−ΔCt^ method was used to calculate relative expression.

### Flow cytometry and antibodies

Total splenocytes were processed and filtered through a 40-µm cell strainer and subjected to RBC lysis (0.86% NH_4_Cl solution) to create single-cell suspensions for flow cytometry analysis. Cells were maintained in complete RPMI (RPMI 1640, 10% fetal plex, 10% non-essential amino acids, 10% sodium pyruvate, 10% L-glutamine, 10% penicillin and streptomycin, and 1% 2-β-mercaptoethanol). To prevent non-specific antibody (Ab) binding to Fc receptors on cells, these receptors were blocked with anti-mouse CD16/32 (clone 24G.2; BioXCell) in fluorescence-activated cell sorting (FACS) buffer containing normal mouse and rat IgG (Life Technologies). FACS buffer (1 × PBS, 0.2% bovine serum albumin, and 0.2% 0.5 M EDTA) was used for washing cells. Flow cytometry Abs are listed in Table SII2 and were prepared in FACS buffer supplemented with Super Bright Staining Buffer (Thermo Fisher) for surface staining any panels containing Abs conjugated to Brilliant Violet fluorophores. Upon completion of surface staining, cells were fixed in 4% paraformaldehyde (Electron Microscopy Sciences) if intracellular staining was not required. Fluorescence minus one (FMO) controls were used to set positive gates. An LSRIIFortessa (Becton Dickson) was used to acquire samples. Data were analyzed using FlowJo version 10 software.

### Intracellular staining

A Foxp3 staining buffer set (eBioscience) was used as per the manufacturer’s direction for intracellular staining of transcription factors. For intracellular cytokine staining, processed splenocytes were first incubated with phorbol 12-myristate 13-actetate (PMA; 0.1 µg/mL) and ionomycin (1 µg/mL) in the presence of Brefeldin A (BFA; 20 µg/mL) (Sigma) at 37°C for 4 hours to stimulate cytokine production. After this incubation period, cells underwent surface staining followed by fixation with 4% paraformaldehyde and permeabilization with 0.1% saponin diluted in FACS buffer. Cells were then subjected to cytokine staining with IFN-γ, IL-10, TNF, and GM-CSF in the permeabilization buffer. FMO controls were used to set positive gates. Antibodies used for flow cytometry are listed in Table S2.

### Adoptive transfer experiments

Donor naïve (CD44^lo^CD62L^hi^) CD4^+^ T cells were purified and sorted from the spleen of naïve PbT-II and *Bhlhe40^−/^* ^−^ PbT-II Tg mice on a CD45.2 background. A total of 10^4^ wild-type (WT) or *Bhlhe40^−/^* ^−^ PbT-II T cells were transferred retro-orbitally into recipient CD45.1^+^ mice. One day later, recipient mice were challenged with 10^5^ *P. yoelii* 17X pRBCs i.p. Splenocytes were assessed for cytokine production on day 7 p.i. following *ex vivo* restimulation with PMA and ionomycin in the presence of BFA, as described above. Donor Tg T cells were identified by flow cytometry based on dual staining with fluorescently labeled Abs against CD45.2 and TCRVα2, and cytokine production was assessed by intracellular staining as described above.

To evaluate T-cell proliferation, donor splenocytes from naïve C57BL/6J WT and *Bhlhe40^−/^* ^−^ mice were labeled with 10 µM of carboxyfluorescein succinimidyl ester (CFSE; Sigma). After CFSE labeling, CD4^+^ T cells were enriched using anti-CD4 microbeads (Miltenyi Biotec) and an AutoMACS Pro cell separator (Miltenyi Biotech). Afterward, 2.5 × 10^5^ donor cells were transferred intravenously into recipient Thy1.1^+^ mice. Mice were subsequently infected 1 day after cell transfer, and donor cells were recovered 5 days after infection.

### Cytokine enzyme-linked immunosorbent assays (ELISAs)

Serum IL-10 and IFN-γ were quantified using sandwich ELISA. Plates were coated overnight with anti-IL-10 (Clone JES5-2A5, BioLegend) or anti-IFN-γ (Clone AN-18, BioLegend) Ab at 4°C in PBS. Serum samples were used neat, while protein standards were diluted in complete Roswell Park Memorial Institute 1640 media and added to plates. The plates were incubated at 37°C for 2 hours, washed, and then incubated with biotinylated anti-IL-10 (Clone JES5-16E3, BioLegend) or anti-IFN-γ (Clone R4-6A2, BioLegend) for 1 hour at room temperature. Next, Streptavidin-HRP (Jackson ImmunoResearch) was added to each plate after washing and incubated at room temperature for 30 minutes. For detection of IL-10, SureBlue substrate (KPL) was used with stop solution (KPL) added to stop the enzymatic reaction, and plates were read at an absorbance of 450 nm on a FLUOStar Omega plate reader (BMG Labtech). To detect IFN-γ, ABTS (KPL) was used, and plates were read at an absorbance of 405 nm. For recall assays, splenocytes were plated in triplicate at a concentration of 5 × 10^5^ cells/well in a final volume of 200 µL in a 96-well round-bottom plate (Genesee Sci). Cells were left unstimulated or were stimulated with 1 µg/mL anti-CD3e or 10 µg/mL of soluble red blood cell-derived *Plasmodium* lysate. Cells were incubated at 37°C for 48 hours, and the supernatant was used to measure IL-10 and IFN-γ by ELISA.

### Antibody ELISAs

Recombinant *P. yoelii* AMA-1 or MSP-1_19_ proteins diluted in sodium bicarbonate buffer were used to coat high-binding Immunlon HBX plates (Thermo Scientific) at 4°C overnight. First, plates were washed 5× in PBS + Tween buffer between each step. Next, plates were blocked with 5% fetal bovine serum (Life Technologies) in PBS for 2 hours at 37°C. The serum was diluted at 1:50 initially and then serially 1:3 down the plate; afterward, the plates were incubated for 2 hours at 37°C. HRP-conjugated IgM or IgG (Southern Biotech) was then added to the plates and incubated at 37°C for 1 hour. Finally, SureBlue substrate (KPL) was added for detection, and each plate was exposed for 15 minutes before adding the stop solution (KPL). Plates were read at an absorbance of 450 nm on a FLUOStar Omega plate reader (BMG Labtech).

### α-IL-10R blocking

Wild-type C57BL/6J mice were administered 200 µg of α-IL-10R (1B1.3A; BioXCell) in sterile PBS i.p. on days 6, 9, and 12 p.i. α-Rat IgG (Sigma) was administered as a control. *Il10^−/^* ^−^ mice were utilized as controls.

### Statistics

GraphPad Prism 9 (GraphPad Software, Inc., San Diego, CA) and R 3.4.3 (The R Foundation) were used for statistical analyses. Statistical significance was determined using a nonparametric Mann-Whitney *t*-test for a single comparison between groups. For multiple comparisons between groups, statistical significance was determined by a one-way analysis of variance (ANOVA) with a post-Kruskal-Wallis multiple comparisons test or a two-way ANOVA with a post hoc Holm-Sidak multiple-comparison test. A *P*-value of <0.05 was considered statistically significant. The figure legends provide additional details about specific tests of statistical analysis.

## RESULTS

### Bhlhe40 expression is required to control a *P. yoelii* 17X infection in mice efficiently

**Fig 1:**
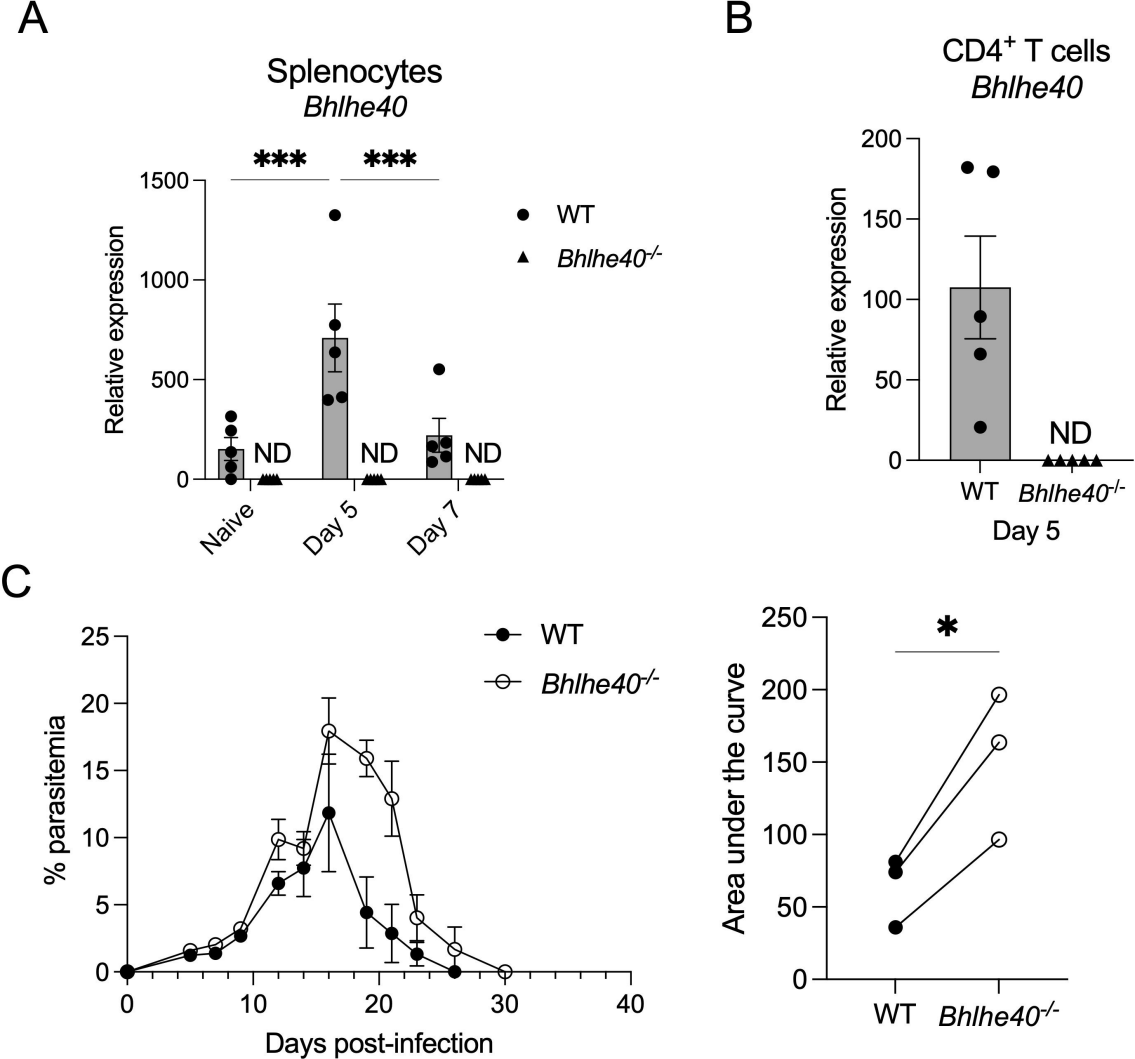
Bhlhe40 expression is required to control a non-lethal *P. yoelii* 17X infection in mice efficiently. WT and *Bhlhe40^−/^* ^−^ mice were infected i.p. with 10^5^ *P. yoelii* pRBCs. Relative expression of *Bhlhe40* in (**A**) total splenocytes from naïve mice and at days 5 and 7 p.i. and (**B**) sort-purified CD4^+^ T cells isolated from the spleen at day 5 p.i. as determined by RT-qPCR. Data were normalized to *Hprt*, and the 2^−ΔCt^ method was used to calculate relative expression. (**C**) Representative parasitemia curve during primary infection as determined by flow cytometry. The area under the curve was measured for WT and *Bhlhe40^−/^* ^−^ mice from the day of peak parasitemia to the day of parasite clearance in WT mice from three separate experiments. RT-qPCR data represent two independent experiments with three to five mice per group. Parasitemia was assessed as part of three independent experiments with at least five mice per group. (**A**) A nonparametric Mann-Whitney *t*-test determined significance. (**C**) A paired *t*-test determined significance. *P < 0.05, ***P < 0.001. ND, not detected.

The transcription factor Bhlhe40 promotes protective immune responses in mice against the Th1-centric pathogens *T. gondii* and *M. tuberculosis* (18, 19). Therefore, it was of interest to determine if Bhlhe40 plays a similar role in response to infection with non-lethal *P. yoelii* 17X. First, RNA was isolated from the spleen of naïve and infected mice to determine if Bhlhe40 is expressed in immune cells in response to *P. yoelii* infection. Transcripts for *Bhlhe40* were present in the spleen of naïve WT mice and after infection, with a significant increase in expression seen at day 5 p.i. (Fig. 1A). However, the increase in *Bhlhe40* expression was short lived as it declined at day 7 p.i. Specificity was confirmed by the lack of detected *Bhlhe40* RNA in the spleen of *Bhlhe40^−/^* ^−^ mice. Next, given the role of Bhlhe40 expression in CD4^+^ T cells in contributing to the control of *M. tuberculosis* and *T. gondii* infections in mice (18, 19), the expression of Bhlhe40 RNA was confirmed in purified CD4^+^ T cells isolated from the spleen of WT mice after *P. yoelii* infection (Fig. 1B). Again, specificity was established by the lack of detected *Bhlhe40* RNA in CD4^+^ T cells from *Bhlhe40^−/^* ^−^ mice.

Since Bhlhe40 was expressed during infection, the ability of *Bhlhe40^−/^* ^−^ mice to control a *P. yoelii* infection was monitored. *Bhlhe40^−/^* ^−^ mice exhibited a significant difference in parasite burden from peak parasitemia through clearance compared to WT mice (Fig. 1C). Thus, these data suggest that Bhlhe40 is an important transcription factor contributing to the control of infection with *P. yoelii* in mice.

### IL-10 production is enhanced in *Bhlhe40^−/^* ^−^ mice during the early stages of *P. yoelii* infection

**Fig 2:**
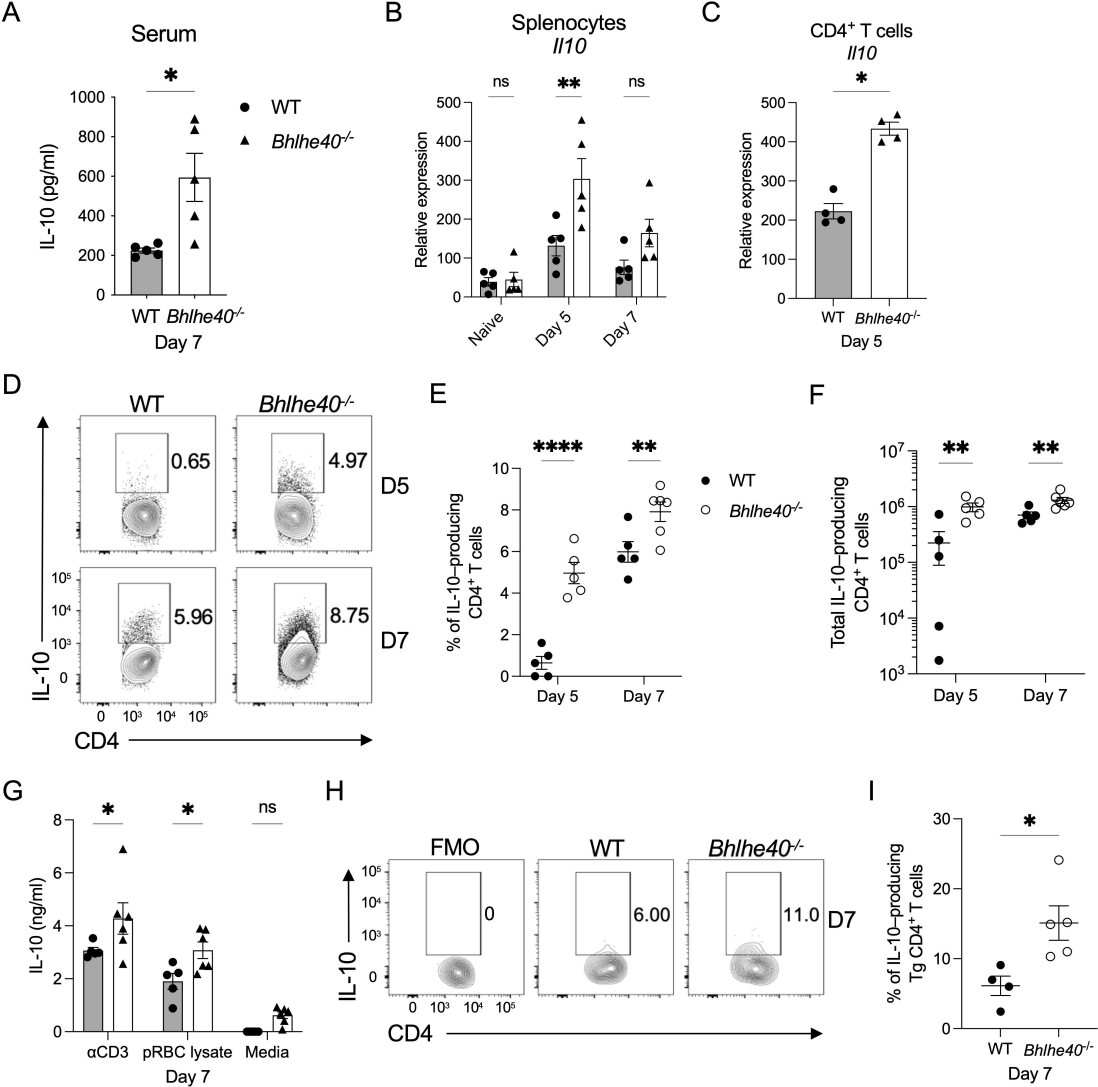
IL-10 production is enhanced in *Bhlhe40^−/^* ^−^ mice after *P. yoelii* infection. WT and *Bhlhe40^−/^* ^−^ mice were infected i.p. with 10^5^ *P. yoelii* pRBCs. (**A**) Serum concentrations of IL-10 at day 7 p.i. as determined by ELISA. Relative expression of *Il10* in (**B**) total splenocytes from naïve mice and mice infected for 5 and 7 days and (**C**) sort-purified CD4^+^ T cells isolated from the spleen at day 5 p.i. as determined by RT-qPCR. Data were normalized to *Hprt*, and the 2^−ΔCt^ method was used to calculate relative expression. (**D**) Representative flow plots of live, single-cell, IL-10–producing CD4^+^ T cells isolated from the spleen at days 5 and 7 p.i. following *ex vivo* stimulation with PMA and ionomycin in the presence of BFA. Gate based on FMO control displayed in Fig. S1A. Frequency (**E**) and total number (**F**) of live, single-cell IL-10–producing CD4^+^ T cells at days 5 and 7 p.i. (**G**) Concentration of IL-10 measured by ELISA in the supernatant of WT and *Bhlhe40^−/^* ^−^ splenocytes isolated at day 7 p.i. and cultured in the presence of anti-CD3e, parasite-derived lysate or media alone for 48 hours. (**H**) Representative flow plots of live, single-cell, CD45.2^+^TCRVα2^+^ IL-10–producing WT or *Bhlhe40^−/^* ^−^ donor PbT-II Tg CD4^+^ T cells recovered from the spleen of CD45.1^+^ recipient mice at day 7 p.i. following *ex vivo* stimulation with PMA and ionomycin in the presence of BFA. (**I**) Frequency of live, singlet IL-10–producing WT or *Bhlhe40^−/^* ^−^ PbT-II Tg CD4^+^ T cells at day 7 p.i. Gate based on FMO control. (**A and G**) Data represent two independent experiments with five to six mice per group. (**B and C**) Data represent two independent experiments with three to five mice per group. (**D–F**) Data represent three independent experiments with five to six mice per group. (**H and I**) Data represent three independent experiments with four to five mice per group. (**A, C, and I**) A nonparametric Mann-Whitney *t*-test determined significance. (**B and E–G**) A two-way ANOVA determined significance with a post hoc Holm-Sidak’s multiple-comparison test. *P < 0.05, **P < 0.01, ****P < 0.0001, ns, not significant.

A principal function of Bhlhe40 in infectious and autoimmune disease models in mice is to suppress IL-10 expression in CD4^+^ T cells (17 – 19, 22). Thus, it was of interest to determine if the increase in parasite burden and lag in parasite clearance observed in *Bhlhe40^−/^* ^−^ mice was due to elevated IL-10 production, particularly by CD4^+^ T cells, given their role in IL-10 production in response to this infection (23). By day 7 p.i., the quantity of IL-10 in the serum was significantly elevated in the *Bhlhe40^−/^* ^−^ mice (Fig. 2A), suggesting an early role for Bhlhe40 in suppressing IL-10 expression after infection. Increased serum concentrations of IL-10 correlated with a higher expression of *Il10* mRNA in the spleen of *Bhlhe40^−/^* ^−^ mice compared to WT mice on days 5 and 7 p.i. (Fig. 2B). Furthermore, *Il10* mRNA expression was significantly elevated in purified CD4^+^ T cells isolated from the spleen of *Bhlhe40^−/^* ^−^ mice at day 5 compared to WT CD4^+^ T cells (Fig. 2C). When protein expression was examined after *ex vivo* restimulation, a significantly higher frequency and number of splenic CD4^+^ T cells from *Bhlhe40^−/^* ^−^ mice produced IL-10 compared to their WT counterparts at days 5 and 7 (Fig. 2D through F; Fig. S1A). Consistent with this observation, splenocytes from *Bhlhe40^−/^* ^−^ mice stimulated with anti-CD3e or parasite-derived lysate produced more IL-10 than the WT splenocytes (Fig. 2G).

Lastly, to confirm that the increased production of IL-10 by CD4^+^ T cells was occurring in an antigen-specific manner, PbT-II CD4^+^ T cells (20) crossed onto a Bhlhe40-deficient background (*Bhlhe40^−/^* ^−^ PbT-II mice) were adoptively transferred into congenic recipients that were subsequently infected with *P. yoelii*. Compared to WT PbT-II cells, more of the recovered donor *Bhlhe40^−/^* ^−^ PbT-II T cells produced IL-10 (Fig. 2H and I). Overall, the data suggest that Bhlhe40 negatively regulates IL-10 production in the spleen after infection with *P. yoelii* and that splenic antigen-specific CD4^+^ T cells contribute to the elevated IL-10 production in the absence of Bhlhe40.

### The loss of Bhlhe40 does not impair local production of IFN-γ by splenic CD4 T cells

**Fig 3:**
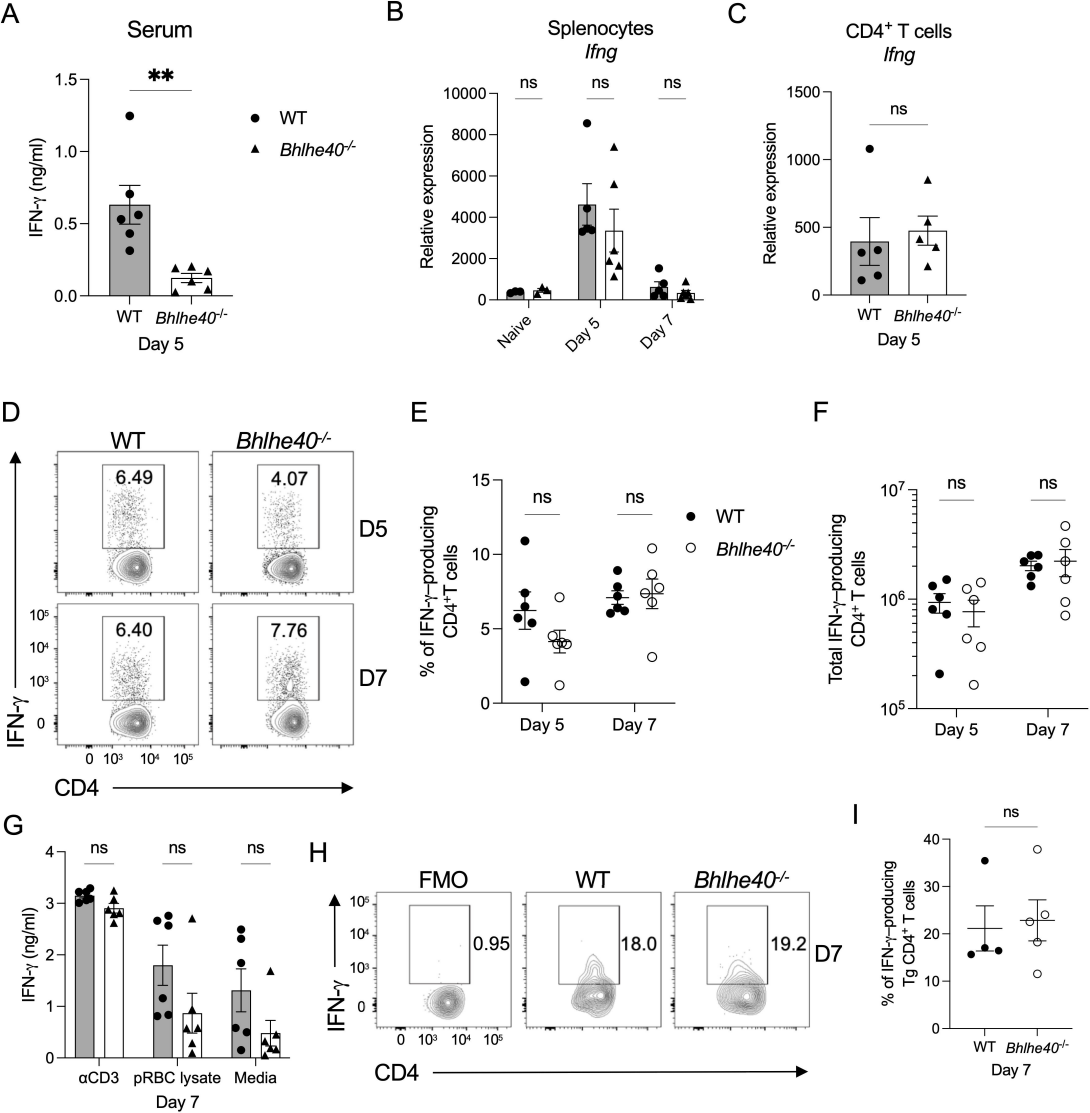
*Ifng* expression by splenic CD4^+^ T cells is not impaired after *P. yoelii* infection. WT and *Bhlhe40^−/^* ^−^ mice were infected i.p. with 10^5^ *P. yoelii* pRBCs. (**A**) Serum concentrations of IFN-γ at day 5 p.i. as determined by ELISA. Relative expression of *Ifng* in (**B**) total splenocytes from naïve mice and mice infected for 5 and 7 days and (**C**) sort-purified CD4^+^ T cells isolated from the spleen at day 5 p.i. as determined by RT-qPCR. Data were normalized to *Hprt*, and the 2^−ΔCt^ method was used to calculate relative expression. (**D**) Representative flow plots of live, single-cell, IFN-γ–producing CD4^+^ T cells isolated from the spleen at days 5 and 7 p.i. following *ex vivo* stimulation with PMA and ionomycin in the presence of BFA. Gate based on FMO control displayed in Fig. S1A. Frequency (**E**) and total number (**F**) of live, singlet IFN-γ–producing CD4^+^ T cells isolated from the spleen at days 5 and 7 p.i. (**G**) Concentration of IFN-γ measured by ELISA in the supernatant of WT and *Bhlhe40^−/^* ^−^ splenocytes isolated at day 7 p.i. and cultured in the presence of anti-CD3e, parasite-derived lysate or media alone for 48 hours. (**H**) Representative flow plots of live, single-cell, CD45.2^+^TCRVα2^+^ IFN-γ–producing donor WT or *Bhlhe40^−/^* ^−^ PbT-II Tg CD4^+^ T cells recovered from the spleen of CD45.1^+^ recipient mice at day 7 p.i. following *ex vivo* stimulation with PMA and ionomycin in the presence of BFA. Gate based on FMO control. (**I**) Frequency of live, singlet IFN-γ–producing WT or *Bhlhe40^−/^* ^−^ PbT-II Tg CD4^+^ T cells at day 7 p.i. (**A, B, and G**) Data represent two independent experiments with five to six mice per group. (**C**) Data represent two independent experiments with three to five mice per group. (**D–F**) Data represent three independent experiments with five to six mice per group. (**H and I**) Data represent three independent experiments with four to five mice per group. (**A, C, and I**) A nonparametric Mann-Whitney *t*-test determined significance. (**B and E–G**) A two-way ANOVA determined significance with a post hoc Holm-Sidak’s multiple-comparison test. **P < 0.01, ns, not significant.

IFN-γ plays a crucial role in parasite clearance (24, 25), and Bhlhe40 is suggested to promote IFN-γ expression (19, 26). Thus, IFN-γ expression was examined during *P. yoelii* infection in WT and *Bhlhe40^−/^* ^−^ mice. By day 5 p.i., the quantity of IFN-γ in the serum was significantly impacted by the loss of Bhlhe40 expression (Fig. 3A). However, this difference was short lived as IFN-γ was below the limit of detection by day 7 in the serum of WT and Bhlhe40 mice (data not shown). Examination of *Ifng* gene expression in the spleen after infection did not reveal a significant difference in the expression of this gene between WT and *Bhlhe40^−/^* ^−^ splenocytes or splenic CD4^+^ T cells after infection (Fig. 3B and C). Furthermore, T-bet, a transcription factor that promotes IFN-γ expression by CD4^+^ T cells and can interact with Bhlhe40 in NKT cells to regulate IFN-γ expression (27), showed no significant difference in expression between WT and *Bhlhe40^−/^* ^−^ splenocytes or splenic CD4^+^ T cells after infection (Fig. S1B and C), although the expression of *Tbx21* was increased in the spleen of *Bhlhe40^−/^* ^−^ mice 7 days after infection compared to day 5.

Likewise, examination of IFN-γ protein production after restimulation did not indicate that splenic *Bhlhe40^−/^* ^−^ CD4^+^ T cells are deficient in producing IFN-γ (Fig. 3D through F; Fig. S1A). Nor is T-bet protein expression impacted by the loss of Bhlhe40 (Fig. S1D and E). Furthermore, no defect in IFN-γ production by CD8^+^ T cells was seen after infection (Fig. S1F through H). While restimulation of splenocytes with anti-CD3e or parasite-derived lysate did not reveal a significant defect in IFN-γ secretion in the absence of Bhlhe40 (Fig. 3F). However, there was a trend toward a reduction in IFN-γ secretion by the *Bhlhe40^−/^* ^−^ splenocytes in response to the parasite lysate. Lastly, the adoptive transfer experiments with WT and *Bhlhe40^−/^* ^−^ PbT-II cells indicated that a similar proportion of these Ag-specific T cells produced IFN-γ after infection (Fig. 3G and H).

Given the prominent role of IL-10^+^IFN-γ^+^ type 1 regulatory T (Tr1) cells in suppressing inflammation in response to *Plasmodium* infection in mice and humans (28 – 30), the Tr1 cell population was examined in the spleen. Significantly more Tr1 cells were present in the spleen of *Bhlhe40^−/^* ^−^ mice than WT mice at day 5 p.i. (Fig S1I through K). While there was no difference in the proportion of Tr1 cells at day 7 p.i., their numbers were still significantly higher in the *Bhlhe40^−/^* ^−^ mice. Lastly, examination of cytokine production at later times after infection indicated that CD4^+^ T cells did not maintain their ability to produce IL-10 at a higher rate in the absence of Bhlhe40 (Fig S2). Overall, these data suggest that Bhlhe40 acts as an early negative regulator of IL-10 expression in CD4^+^ T cells within the spleen after *P. yoelii* infection. Also, these results indicate that the absence of Bhlhe40 does not impede local production of IFN-γ by CD4^+^ and CD8^+^ T cells in the spleen after *P. yoelii* infection, although systemic production of IFN-γ is affected by the loss of Bhlhe40.

### Bhlhe40 is not required for CD4^+^ T-cell proliferation during early *P. yoelii* infection

**Fig 4:**
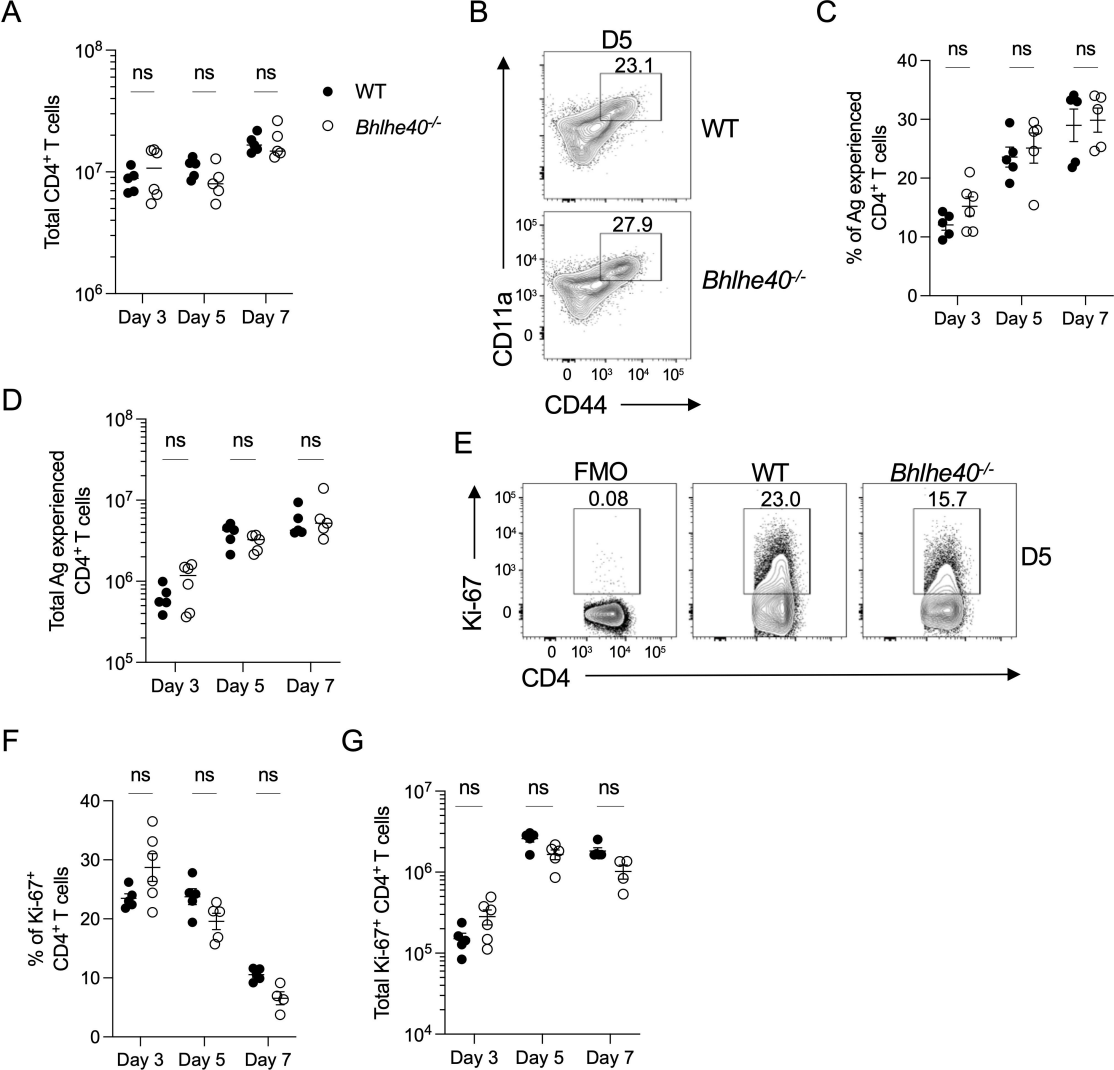
Bhlhe40 is not required for CD4^+^ T-cell proliferation during *P. yoelii* infection. WT and *Bhlhe40^−/^* ^−^ mice were infected i.p. with 10^5^ *P. yoelii* pRBCs. (**A**) The total number of live, singlet CD4^+^ T cells in WT and *Bhlhe40^−/^* ^−^ mice infected for 3, 5, and 7 days. (**B**) Representative flow plot of live, singlet CD44^hi^CD11a^+^ CD4^+^ T cells from WT and *Bhlhe40^−/^* ^−^ mice at day 5 p.i. Frequency (**C**) and total number (**D**) of live, singlet CD44^hi^CD11a^+^ CD4^+^ T cells from mice infected for 3, 5, and 7 days. (**E**) Representative flow plots of Ki-67 expression by live, singlet CD44^hi^CD11a^+^ CD4^+^ T cells isolated from the spleen of mice infected for 5 days. Gate based on FMO control. Frequency (**F**) and total number (**G**) of live, singlet CD44^hi^CD11a^+^ Ki-67^+^ CD4^+^ T cells from the spleen of WT and *Bhlhe40^−/^* ^−^ mice infected for 3, 5, and 7 days. Data represent two independent experiments with four to five mice per group. A two-way ANOVA determined significance with a post hoc Holm-Sidak’s multiple-comparison test. ns, not significant.

Besides acting as a regulator of cytokine expression, Bhlhe40 is also linked to promoting T-cell proliferation (31). To determine if T-cell proliferation is impacted by the loss of Bhlhe40 during *Plasmodium* infection, Ki-67 was utilized as a surrogate marker of proliferation. When CD4^+^ T cells were examined for differences in numbers and activation, no differences in total CD4^+^ T cells or antigen-experienced CD4^+^ T cells were apparent in the spleen of infected *Bhlhe40^−/^* ^−^ and WT mice (Fig. 4A through D). Analysis of Ki-67 expression revealed no significant differences in the frequency or the total number of Ki-67^+^ CD4^+^ T cells in infected *Bhlhe40^−/^* ^−^ mice (Fig. 4E through G). Furthermore, the adoptive transfer of CFSE-labeled donor CD4^+^ T cells did not reveal any defect in the ability of the Bhlhe40-deficient CD4^+^ T cells to proliferate in response to *P. yoelii* infection (Fig. S3A and B). Together, these data suggest that Bhlhe40 is not required to promote CD4^+^ T-cell accumulation and proliferation in the spleen during *P. yoelii* infection.

### The absence of Bhlhe40 does not impact Treg cell numbers in the spleen

Bhlhe40 contributes to Treg cell homeostasis by promoting their survival and expansion in the periphery (32). To determine if the loss of Bhlhe40 impacts Treg accumulation in the spleen, CD25^+^Foxp3^+^ Treg cell numbers were evaluated in the spleen before and after infection. No significant difference in the proportion or number of CD25^+^Foxp3^+^ Treg cells was apparent in the spleen of naïve WT and *Bhlhe40^−/^* ^−^ mice (Fig. S3C through E). While a significant difference in the frequency of Tregs in the spleen was observed at day 5 p.i., this did not equate to a difference in Treg numbers. Furthermore, no difference in the proportion or number of Tregs was seen on day 7. These data suggest that Treg accumulation in the spleen before and after infection is not affected by the loss of Bhlhe40.

### *Bhlhe40^−/^* ^−^ mice generate a robust humoral response

Abs and B cells are crucial in controlling infection with *P. yoelii* in mice (33, 34). Abs are produced via GC-independent and GC-dependent means in the spleen in response to *Plasmodium* infection and rely on Tfh cells (35 – 37). Given the contribution of IL-10 in constricting the Tfh cell response after *P. yoelii* infection (29), we predicted that the Tfh cell and B-cell responses would be reduced without Bhlhe40. However, Tfh cell accumulation in the spleen was similar between WT and *Bhlhe40^−/^* ^−^ mice after infection (Fig. S4A through C). Also, early plasmablast expansion and GC B-cell accumulation were not impacted by the loss of Bhlhe40 expression (Fig. S4D through I), suggesting that the humoral response is intact in the absence of Bhlhe40. Indeed, no significant differences in Ab titers were observed against AMA-1 and MSP-1_19_ in *Bhlhe40^−/^* ^−^ mice compared to WT mice at day 23 p.i. (Fig. S4J). As the binding affinity of Ab increases as the GC reaction progresses, the affinity maturation of Ag-specific Abs from WT and *Bhlhe40^−/^* ^−^ mice was evaluated to determine if a loss of Bhlhe40 impacted GC function. There was a slight but non-significant increase in the affinity of AMA-1–specific IgG observed in the *Bhlhe40^−/^* ^−^ mice compared to WT mice, but no difference in IgG Ab affinity for MSP-1_19_ was detected at day 23 p.i. (Fig. S4K). Thus, these results indicate that Bhlhe40 is not required to promote the humoral response after *P. yoelii* infection, nor did the increase in IL-10 production inhibit the development of the humoral response.

### Blockade of IL-10R signaling reduces parasite burden in WT and *Bhlhe40^−/^* ^−^ mice after *P. yoelii* 17X infection

**Fig 5:**
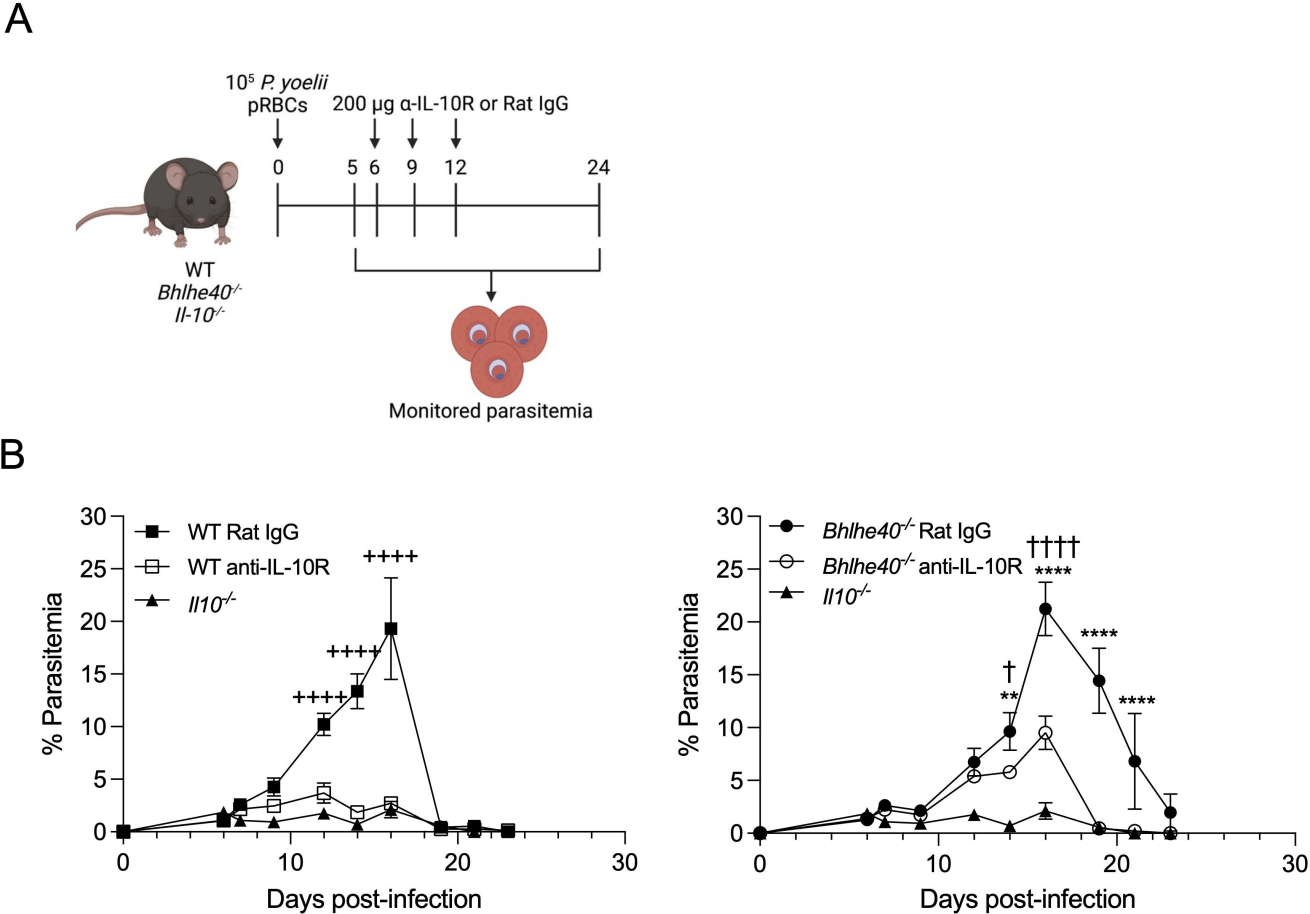
Blockade of IL-10R signaling reduces parasite burden in WT and *Bhlhe40^−/^* ^−^ mice after *P. yoelii* 17X infection. (**A**) Experimental design. *Bhlhe40^−/^* ^−^ and WT mice were administered 200 µg of an anti-IL-10R Ab or a rat IgG isotype control Ab i.p. on days 6, 9, and 12 p.i. with *P. yoelii* 17X. *Il10^−/^* ^−^ mice were also infected and served as a control. (**B**) Representative parasitemia curve as determined by flow cytometry. Data are representative of three independent experiments with five mice per group. Two-way ANOVA determined significance with a post hoc Holm-Sidak multiple-comparison test. (+) WT Rat IgG vs WT anti-IL-10R, (*) *Bhlhe40^−/^* ^−^ Rat IgG vs *Bhlhe40^−/^* ^−^ anti-IL-10R, (†) WT anti-IL-10R vs *Bhlhe40^−/^* ^−^ anti-IL-10R. One symbol, *P* < 0.05; two symbols, *P* < 0.01; four symbols, *P* < 0.0001. Panel A created with BioRender.com.

Since IL-10 production was increased in *Bhlhe40^−/^* ^−^ mice and given the finding that blocking IL-10R signaling reversed the susceptibility phenotype seen after *T. gondii* infection (19), an anti-IL-10R Ab was used to determine if IL-10 blockade could improve the ability of the *Bhlhe40^−/^* ^−^ mice to control and clear their infection with *P. yoelii*. As shown in the experimental design schema in Fig. 5, WT, *Bhlhe40^−/^* ^−^, and *Il10^−/^* ^−^ mice were infected with *P. yoelii* 17X. Groups of WT and *Bhlhe40^−/^* ^−^ mice were administered either an α-IL-10R or Rat IgG isotype control Ab on days 6, 9, and 12 p.i. (Fig. 5A). The timing of the Ab treatment was based on findings showing blockade of IL-10R signaling at an earlier time after infection results in a defect in the B-cell response to *P. yoelii* (13).

Parasite burden in WT mice treated with α-IL-10R Abs decreased the parasite burden to amounts similar to that seen in *Il10^−/^* ^−^ mice, suggesting that blockade of IL-10R in WT mice was sufficient to improve infection control (Fig. 5B). IL-10R blockade also significantly decreased parasite burden in *Bhlhe40^−/^* ^−^ mice, although the Ab blockade was not as effective at reducing parasite burden as in the WT mice (Fig. 5B). However, there was no delay in the clearance of the infection with anti-IL-10R treatment in the *Bhlhe40^−/^* ^−^ mice. These data suggest that enhanced IL-10 production plays a significant role in the higher parasite burden and delayed clearance seen in *Bhlhe40^−/^* ^−^ mice after *P. yoelii* infection.

### IFN-γ production by CD4^+^ T cells in the liver is decreased in *Bhlhe40^−/^* ^−^ mice

**Fig 6:**
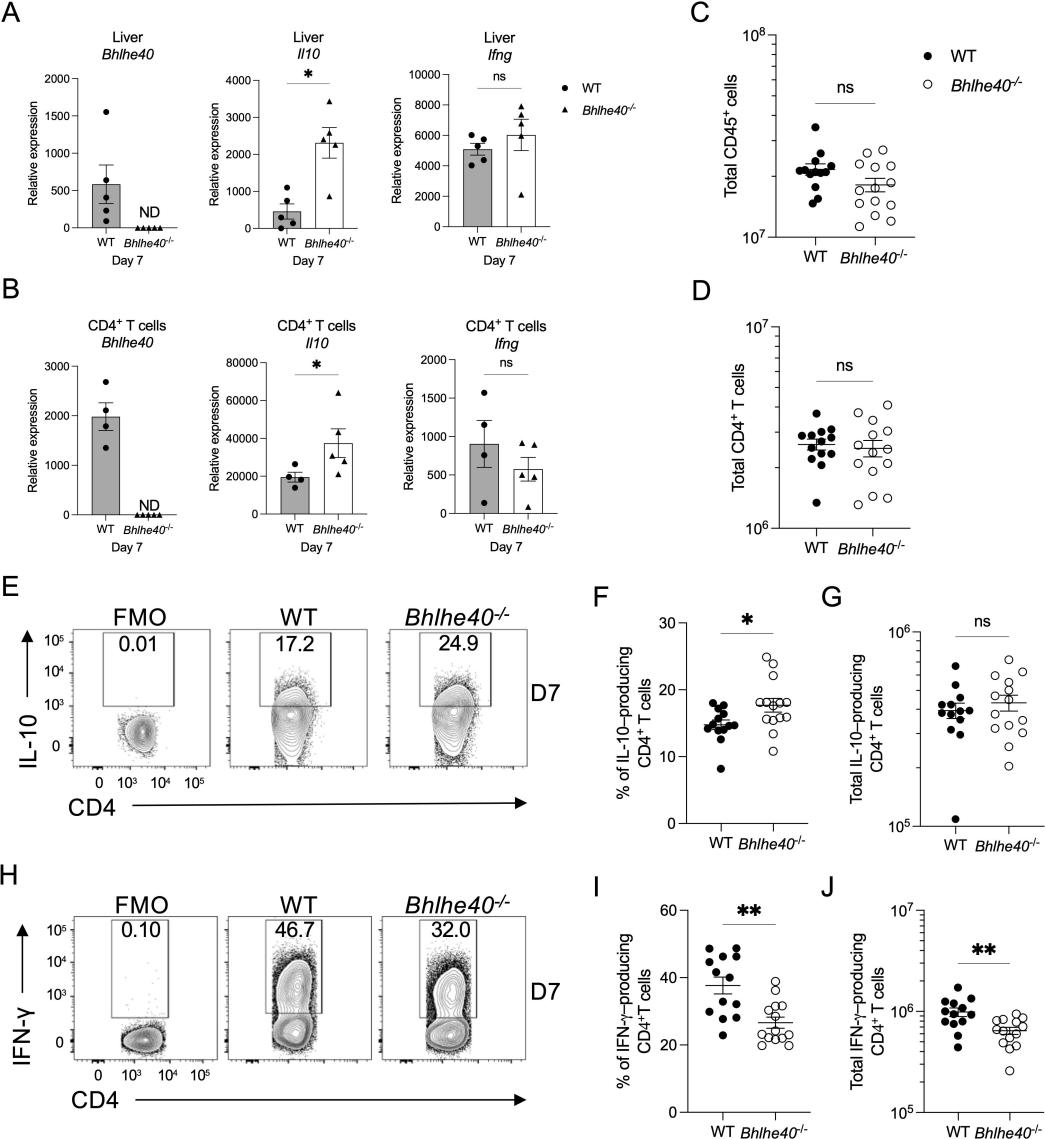
The loss of Bhlhe40 impacts IFN-γ production by CD4^+^ T cells in the liver after *P. yoelii* infection. WT and *Bhlhe40^−/^* ^−^ mice were infected i.p. with 10^5^ *P. yoelii* pRBCs. Relative expression of *Bhlhe40*, *Il10,* and *Ifng* in (**A**) purified CD45^+^ cells and (**B**) sort-purified CD4^+^ T cells isolated from the liver at day 7 p.i. as determined by RT-qPCR. Data were normalized to *Hprt*, and the 2^−ΔCt^ method was used to calculate relative expression. The total number of (**C**) live, singlet CD45^+^ cells (**D**) or CD4^+^ T cells isolated from the liver at day 7 p.i. (**E**) Representative flow plots of IL-10 expression in live, singlet CD4^+^ T cells isolated from the liver at day 7 p.i. following *ex vivo* stimulation with PMA and ionomycin in the presence of BFA. Gate based on FMO control. Frequency (**F**) and the total number of (**G**) live, singlet IL-10–producing CD4^+^ T cells from the liver at day 7 p.i. (**H**) Representative flow plots of IFN-γ expression in live, singlet CD4^+^ T cells isolated from the liver at day 7 p.i. following *ex vivo* stimulation with PMA and ionomycin in the presence of BFA. Gated based on FMO control. (**A and B**) Data represent two independent experiments with five mice per group. (**C–J**) Data are pooled from three independent experiments with three to five mice per group. A nonparametric Mann-Whitney *t*-test determined significance. *P < 0.05; **P < 0.01; ND, not detected. ns, not significant.

After activation in the spleen, T cells can migrate to peripheral tissues, including the liver, during blood-stage infection with *P. yoelii* (23). Furthermore, IL-10–producing CD4^+^ T cells, including Tr1 cells, are abundantly found in the liver and are important for controlling localized inflammation here and in other peripheral tissues (10, 23). To determine if leukocytes express *Bhlhe40* transcripts in the liver during infection with *P. yoelii*, RNA was isolated from liver-derived CD45^+^ cells and CD4^+^ T cells after infection. Indeed, *Bhlhe40* transcripts were detected in leukocytes and purified CD4^+^ T cells isolated from the liver at day 7 p.i. (Fig. 6A and B). The detection of *Bhlhe40* expression in CD4^+^ T cells in the liver suggests a potential role for this transcription factor beyond the initial activation events in T cells that occur in the spleen. Examination of *Il10* and *Ifng* transcripts in the liver indicated a significant increase in *Il10* expression in the *Bhlhe40^−/^* ^−^ mice, while no difference in *Ifng* expression was seen (Fig. 6A). Likewise, *Il10* expression was significantly enhanced in CD4^+^ T cells purified from the liver of *Bhlhe40^−/^* ^−^ mice (Fig. 6B). In contrast, a decrease in *Ifng* expression was observed in the *Bhlhe40^−/^* ^−^ CD4^+^ T cells, but this decrease was not statistically significant (Fig. 6B). These differences in RNA expression were not attributed to disparities in cell recovery or differences in recruitment of T cells to the liver (Fig. 6C and D).

Next, leukocytes from the liver of *P. yoelii* infected WT and *Bhlhe40^−/^* ^−^ mice were restimulated *ex vivo* to assess protein production. *Bhlhe40^−/^* ^−^ mice showed a significant increase in the frequency but not the number of IL-10–producing CD4^+^ T cells in this tissue at day 7 p.i. compared to their WT counterparts (Fig. 6E through G). In contrast to the spleen, IFN-γ–producing CD4^+^ T cells were significantly decreased in frequency and number in the liver of *Bhlhe40^−/^* ^−^ mice at day 7 p.i. (Fig. 6H through J). Furthermore, there was a significant reduction in the number of Tr1 cells in the liver of *Bhlhe40^−/^* ^−^ mice, which was not unexpected given the decrease in IFN-γ–producing CD4^+^ T cells (Fig. S5). Taken together, the data suggest that the systemic increase in IL-10 production plus the systemic decline in IFN-γ, characterized by a reduction in IFN-γ–producing CD4^+^ T cells in peripheral tissues, likely contributed to the increased parasite burden and delay in parasite clearance observed in the *Bhlhe40^−/^* ^−^ mice.

### Bhlhe40 expression in CD4^+^ T cells contributes to the control of infection with *P. yoelii*

**Fig 7:**
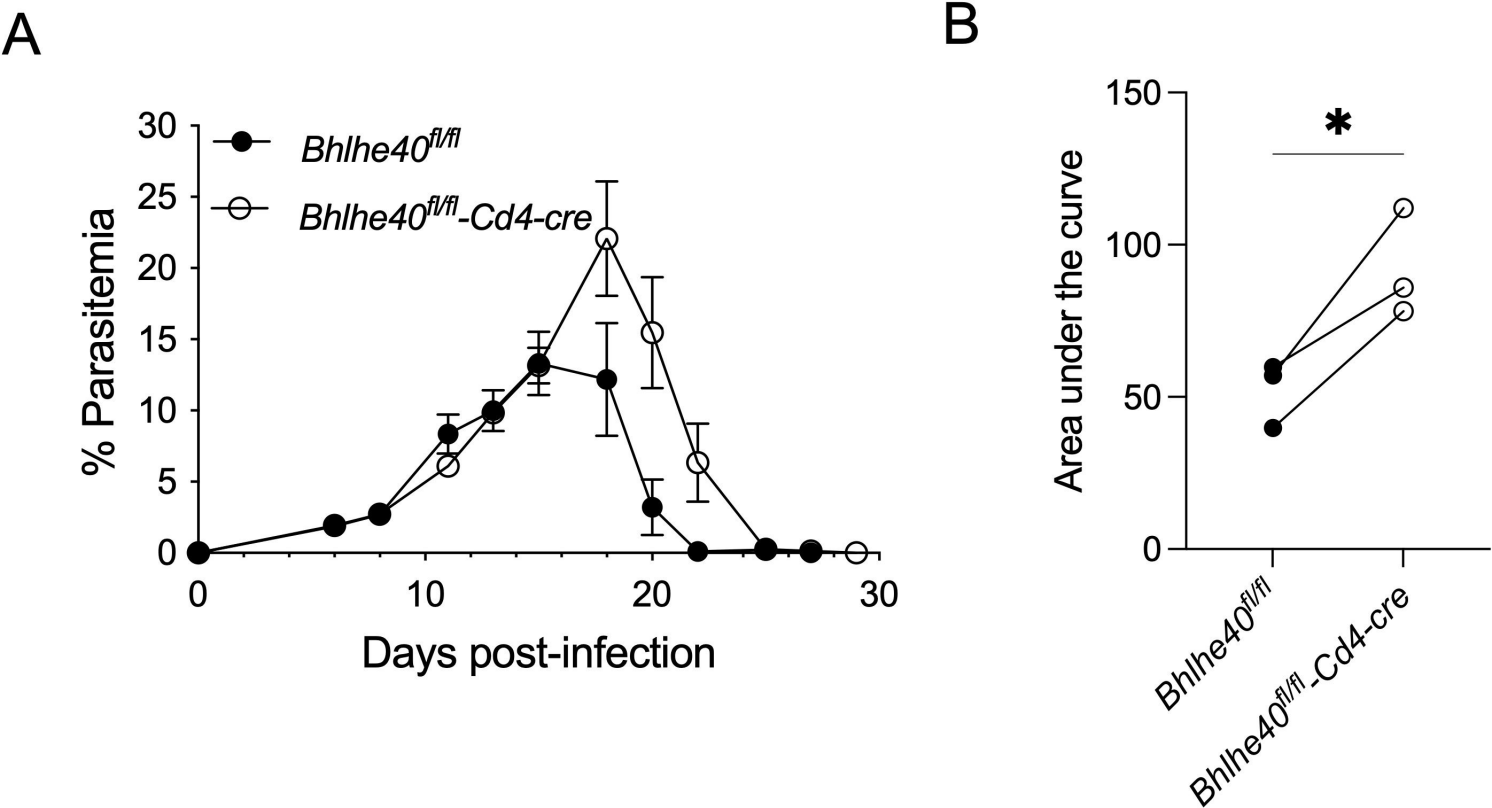
Bhlhe40 expression in CD4^+^ T cells is necessary to control infection with *P. yoelii. Bhlhe40^fl/fl^-Cd4-cre^+^* and *Bhlhe40^fl/fl^ Cd4-cre* ^−^ mice were infected i.p. with 10^5^ *P. yoelii* 17X pRBCs, and parasitemia was monitored throughout the infection, starting at day 5 p.i. (**A**) Representative parasitemia curve as determined by flow cytometry. (**B**) The area under the curve was measured for WT and *Bhlhe40^−/^* ^−^ mice from the day of peak parasitemia to the day of parasite clearance in WT mice from three separate experiments. Data are representative of three independent experiments with five mice per group. A paired *t*-test determined significance. *P < 0.05.

During *P. yoelii* infection, CD4^+^ T cells are essential for controlling parasite burden (33) and are the major producers of IL-10 (23). Furthermore, other infection models have shown that loss of Bhlhe40, specifically in CD4^+^ T cells, results in pronounced disease (18, 19). Thus, it was of interest to determine if the control of a *P. yoelii* infection relied on the expression of Bhlhe40, specifically in T cells. To address this question, *Bhlhe40^fl/fl^-Cd4-cre* mice and littermate *Bhlhe40^fl/fl^* control mice were infected with *P. yoelii*. Similar to their germline knockout counterparts (Fig. 1C), *Bhlhe40^fl/fl^-Cd4-cre* mice showed a significant difference in their ability to control their infection compared to the control *Bhlhe40^fl/fl^* mice (Fig. 7A and B). Also, infection of *Cd4-cre* mice resulted in a parasitemia curve similar to the control *Bhlhe40^fl/fl^* mice (data not shown), indicating that the expression of cre itself did not impact the ability of the mice to control the infection. These data suggest a need for Bhlhe40 expression in T cells to control infection with *P. yoelii* properly. However, these findings do not rule out the contribution of Bhlhe40 expression in additional cell types to the overall phenotype in the *Bhlhe40^−/^* ^−^ mice.

## DISCUSSION

The data presented here demonstrate that the transcriptional regulator Bhlhe40 plays a critical role in supporting the control and clearance of infection with *P. yoelii* in mice. Here, leukocytes, specifically CD4^+^ T cells, upregulate *Bhlhe40* expression in the spleen and liver in response to infection with *P. yoelii*. The expression of Bhlhe40 by CD4^+^ T cells in the spleen during the early days of the acute infection fits with the idea that TCR stimulation and CD28 co-stimulation promote its expression in T cells (17, 38). The presence of Bhlhe40 RNA in CD4^+^ T cells in the liver suggests that expression is maintained after migration or that additional signals in peripheral tissues induce *de novo* expression of Bhlhe40 in antigen-experienced T cells. Nevertheless, the presence of Bhlhe40 RNA in the CD4^+^ T cells isolated from the liver suggests a role for this transcriptional regulator beyond the early stages of T-cell activation in the spleen.

The observation that *Bhlhe40^−/^* ^−^ CD4^+^ T cells exhibit a significant increase in IL-10 RNA and protein production suggests that Bhlhe40 acts during the early stages of a *P. yoelii* infection, to suppress IL-10 expression in CD4^+^ T cells, as it does in response to other Th1-centric infections (18, 19). Hence, by delaying IL-10 expression in T cells and possibly additional cell types, Bhlhe40 allows protective immune responses to operate during a period without strong anti-inflammatory signals, which is critical but not essential for controlling a non-lethal *P. yoelii* infection. Our finding that blockade of IL-10R signaling significantly decreased parasite burden in *Bhlhe40^−/^* ^−^ mice during *P. yoelii* infection supports this conclusion. These findings were similar to those reported with *Bhlhe40^fl/fl^-Cd4-cre* mice, where delivery of α-IL-10R Abs rescued these mice from a lethal outcome with *T. gondii* infection (19).

While IL-10 production by CD4^+^ T cells was elevated without Bhlhe40 after *P. yoelii* infection, this heightened response was short lived. In particular, IFN-γ^+^IL-10^+^ Tr1 cells, which are abundant in the spleen through the contraction phase of the immune response to *P. yoelii* (23, 29, 39), did not show enhanced production of IL-10 past day 7 p.i., nor did they produce more IL-10 in the liver. Perhaps, the activity of Bhlhe40, particularly its ability to suppress *Il10* expression, is limited to the early days of T-cell activation. The rapid decline in Bhlhe40 transcripts in the spleen after infection suggests a transient expression of this gene after T-cell activation, which may be accompanied by a rapid turnover of the transcription factor. The Tr1 cells present at days 5 and 7 in the spleen may represent a population of T cells that have not reached the final stages of maturation, as the majority of CD4^+^ T cells co-express markers such as T-bet/CXCR3 and Bcl6/CXCR5 at this time, suggesting a mixed Th1-Tfh cell phenotype (40 – 42). Therefore, we can speculate that the activity of Bhlhe40, specifically at the *Il10* locus, declines as CD4^+^ T cells mature toward distinct terminally differentiated Th1, Tr1, or Tfh cells.

Instead, at this point (>day 7 p.i.), other factors or mechanisms step in to regulate the production of IL-10 by Tr1 cells and other T cells to prevent sustained overproduction of IL-10 in the absence of Bhlhe40. These regulatory mechanisms could occur at the genomic level or involve post-transcriptional regulation. For instance, IL-10 production is increased in naïve human CD4^+^ T cells in which Bhlhe40 was deleted by a CRISPR/Cas-9 mechanism, but not in total CD4^+^ T cells, although mRNA for IL-10 is increased in both populations (26). This finding suggests that additional post-transcriptional mechanisms can downregulate IL-10 production in CD4^+^ T cells, at least in humans, even in the absence of Bhlhe40. Moreover, post-transcriptional regulation of *Il10* expression could also explain why CD4^+^ T cells in the liver do not produce more IL-10 at this site without Bhlhe40, even though transcripts for IL-10 are significantly increased. Also, other transcription factors could serve to downregulate IL-10 expression at later times after infection; however, the identity of these transcription factors is unclear. IL-10 is also positively regulated by various transcription factors, including Stat1, Stat3, IRF1, BATF, AhR, and c-MAF (7, 43 – 46). Negative regulation of these positive regulators of IL-10 expression in T cells could compensate for the loss of Bhlhe40 as *P. yoelii* infection progresses.

Bhlhe40 is also implicated in regulating *Ifng* expression, although it is unclear if this is through direct or indirect means (15, 18, 19). Here, bulk splenocytes and purified CD4^+^ T cells from *Bhlhe40^−/^* ^−^ mice did not show a significant defect in *Ifng* gene expression, nor was IFN-γ production by Bhlhe40-deficient CD4^+^ T cells impaired following *ex vivo* restimulation with PMA and ionomycin, suggesting that Bhlhe40 does not directly regulate *Ifng* expression or other transcription factors compensate for its loss. In support of this latter idea, *Tbx21* expression was not impacted by the loss of Bhlhe40 after *P. yoelii* infection, suggesting that this transcription factor compensates for any loss in activity of Bhlhe40 at the *Ifng* locus. While Bhlhe40 can serve as a co-factor with T-bet to enhance IFN-γ production in *i*NKT cells (27), the evidence for Bhlhe40-mediated regulation of IFN-γ production in CD4^+^ T cells was shown to be independent of T-bet (19).

Perhaps, given the excess production of IL-10 in the spleen and serum of *Bhlhe40^−/^* ^−^ mice after *P. yoelii* infection accompanied by the defect in systemic IFN-γ and the reduction in IFN-γ secreted by *Bhlhe40^−/^* ^−^ splenocytes restimulated with parasite-derived lysate, Bhlhe40 instead regulates the expression of IFN-γ through an indirect mechanism. Alternatively, Bhlhe40 could modify *Ifng* expression in another cell type in the spleen, although the restimulation assays did not indicate a defect in IFN-γ production by CD8^+^ T cells. However, Bhlhe40 expression may be required for IFN-γ expression by other cell types, such as γδ T cells and NK cells.

In contrast, the data shown here suggest that Bhlhe40 is required to support and maintain *Ifng* expression by CD4^+^ T cells in the periphery during *P. yoelii* infection, as the production of this cytokine was significantly reduced in CD4^+^ T cells from the liver, as opposed to IL-10, whose production was not significantly impacted in the liver in the absence of Bhlhe40. Furthermore, another cytokine whose expression Bhlhe40 is implicated in regulating, GM-CSF (17), was not consistently impacted by the loss of Bhlhe40 expression in T cells in the spleen or liver in this model, neither was TNF (data not shown). Nevertheless, given that IFN-γ is crucial for activating macrophages in response to *Plasmodium* infection, perhaps, the decrease in IFN-γ production by CD4^+^ T cells in the liver negatively impacts the ability of macrophages at this site to clear and kill parasites, contributing to the observed increase in parasite burden in the *Bhlhe40^−/^* ^−^ mice.

Aside from IFN-γ production (24), Ab production is also crucial for *P. yoelii* infection control and clearance (33). The findings here indicate that *Bhlhe40^−/^* ^−^ mice infected with *P. yoelii* exhibit an intact B-cell response, as robust Tfh and GC B-cell populations were observed in *Bhlhe40^−/^* ^−^ mice throughout the infection. Furthermore, the early plasmablast response and parasite-specific Ab titers and Ab affinity maturation were also comparable between *Bhlhe40^−/^* ^−^ and WT mice, which likely explains the ability of the *Bhlhe40^−/^* ^−^ mice to control their infection eventually. However, given that IL-10 can suppress Tfh cell accumulation and parasite-specific Ab responses after *P. yoelii* infection (29) and that Bhlhe40 expression is implicated in restraining Tfh and GC B-cell accumulation (47), it was surprising that the loss of Bhlhe40 did not impact the humoral response. Alternatively, IL-10 is known to support the humoral response to *P. yoelii* through B-cell intrinsic IL-10R signaling (12), resulting in the upregulation of anti-apoptotic proteins, MHC class II and co-stimulatory molecules, and cell-cell adhesion proteins (13). Furthermore, the B-cell promoting activity of IL-10 is prominent in the early stages of B-cell activation before GC formation (13), a timing that matches when IL-10 was observed to be elevated in the spleen of *Bhlhe40^−/^* ^−^ mice. Hence, it is plausible to speculate that the enhanced early production of IL-10 in the *Bhlhe40^−/^* ^−^ mice promoted downstream Ab production through the GC reaction, thus contributing to the eventual control of the parasite.

Although Bhlhe40 is implicated in promoting T-cell proliferation (31), no differences in T-cell proliferation, accumulation, or expression of activation markers were observed in the absence of Bhlhe40 after *P. yoelii* infection, suggesting the early events of T-cell activation are not impaired, matching the findings of others (19, 48). One explanation for these contrasting findings may be due to the background of the *Bhlhe40^−/^* ^−^ mice, as *Bhlhe40^−/^* ^−^ mice on a mixed background were initially utilized to determine the function of Bhlhe40 (31), whereas the *Bhlhe40^−/^* ^−^ mice used here and by others that do not show a proliferative defect are fully backcrossed onto a C57/BL6 background (19, 48). Alternatively, using CD4^+^ T cells derived from another *Bhlhe40^−/^* ^−^ line on a pure C57BL/6 background, another group reported that the Bhlhe40-deficient T cells displayed a proliferative defect *in vitro* in the absence of exogenous IL-2 (38). Taken together, these results indicate that the effects of Bhlhe40 on T-cell proliferation are complex and may be context dependent.

While Bhlhe40 is implicated in Treg homeostasis (32), we did not observe a defect in Treg numbers in the spleen of naïve or *P. yoelii*-infected *Bhlhe40^−/^* ^−^ mice. Furthermore, the defect in Treg numbers was reported in aged mice (>1.2 year), where the mice used here were all between 7 and 12 weeks of age. Furthermore, the Bhlhe40-deficient mice in which the Treg defect was reported were in a separate mouse line (32) from the one used here that does not display the same degree of lymphoproliferative pathology (17). Hence, while a defect in Treg accumulation in the periphery may occur with age in our *Bhlhe40^−/^* ^−^ mice, it did not impact their numbers in this study.

Lastly, while our findings with the *Bhlhe40^fl/fl^-Cd4-cre* mice indicate that Bhlhe40 expression in T cells is critical to controlling infection with *P. yoelii* 17X, this result does not rule out the contribution of Bhlhe40 expression in another cell type to the observed phenotype in the germline knockout mice. One viable cell population that might also require Bhlhe40 expression for infection control is CD11c^+^ cells. In an *M. tuberculosis* infection model, loss of Bhlhe40 expression, specifically in CD11c^+^ cells, resulted in susceptibility of these mice to this infection, similar to what occurs in the *Bhlhe40^fl/fl^-Cd4-cre* mice (18). During a blood-stage *Plasmodium* infection, CD11c^+^ DCs are crucial in initiating and regulating the adaptive immune response (49 – 52). Since significant differences in the adaptive response were not observed beyond day 7, it is reasonable to consider if the innate response is being hampered early in the infection, resulting in a cascade effect that results in a higher parasite burden and a delay in infection resolution. Given that one of the primary functions of IL-10 is to impact antigen-presenting cell (APC) function (53), the excess IL-10 production in the absence of Bhlhe40 expression may serve to primarily dampen APC function at the early stages of the infection both locally in the spleen and peripheral tissues such as the liver. However, this mechanism of action requires further investigation.

While data implicating a role for Bhlhe40 expression in activated macrophages are limited, there is evidence that inflammatory signals can induce Bhlhe40 expression in these cells, suggesting a role for Bhlhe40 in regulating gene expression in macrophages after infection (54). Indeed, Bhlhe40 was shown to promote the expression of genes associated with inflammation, glycolysis, and hypoxia in lipopolysaccharide-stimulated peritoneal macrophages (54). Given the role of macrophages in clearing blood-stage parasites, it is possible that a defect in Bhlhe40-driven gene expression could be a contributing factor to the observed phenotype in *Bhlhe40^−/^* ^−^ mice, although further experiments are warranted.

Overall, the data presented here demonstrate that Bhlhe40 significantly promotes parasite control in response to *P. yoelii* infection. Regulating IL-10 production in CD4^+^ T cells is at least one mechanism by which Bhlhe40 enables parasite control. Further characterization of other immune cell populations in *Bhlhe40^−/^* ^−^ mice will aid in teasing apart the role of this transcriptional regulator in promoting additional anti-parasitic effector mechanisms. Divulging the inner workings of IL-10, specifically how it limits parasite control, and the factors that promote and suppress IL-10 expression during infection is crucial to understanding the immune response to *Plasmodium* infection. Further work in this area will aid in our ability to manipulate these pathways in hopes of creating more efficacious therapeutics and vaccines to treat and prevent malaria or other infections.

## SUPPLEMENTAL MATERIAL

Supplemental material iai.00367-23-s0001.pdf Supplemental figures and tables.

ASM does not own the copyrights to Supplemental Material that may be linked to, or accessed through, an article. The authors have granted ASM a non-exclusive, world-wide license to publish the Supplemental Material files. Please contact the corresponding author directly for reuse.
