# Supplementary material for: Bhlhe40 limits early IL-10 production from CD4+ T cells during Plasmodium yoelii 17X infection
Source: Infect Immun. 2023 Oct 16;91(11):e00367-23. doi: 10.1128/iai.00367-23 (PMC10652903; doi:10.1128/iai.00367-23)
Supplement: Supplemental material — Supplemental figures and tables. [file iai.00367-23-s0001.pdf]

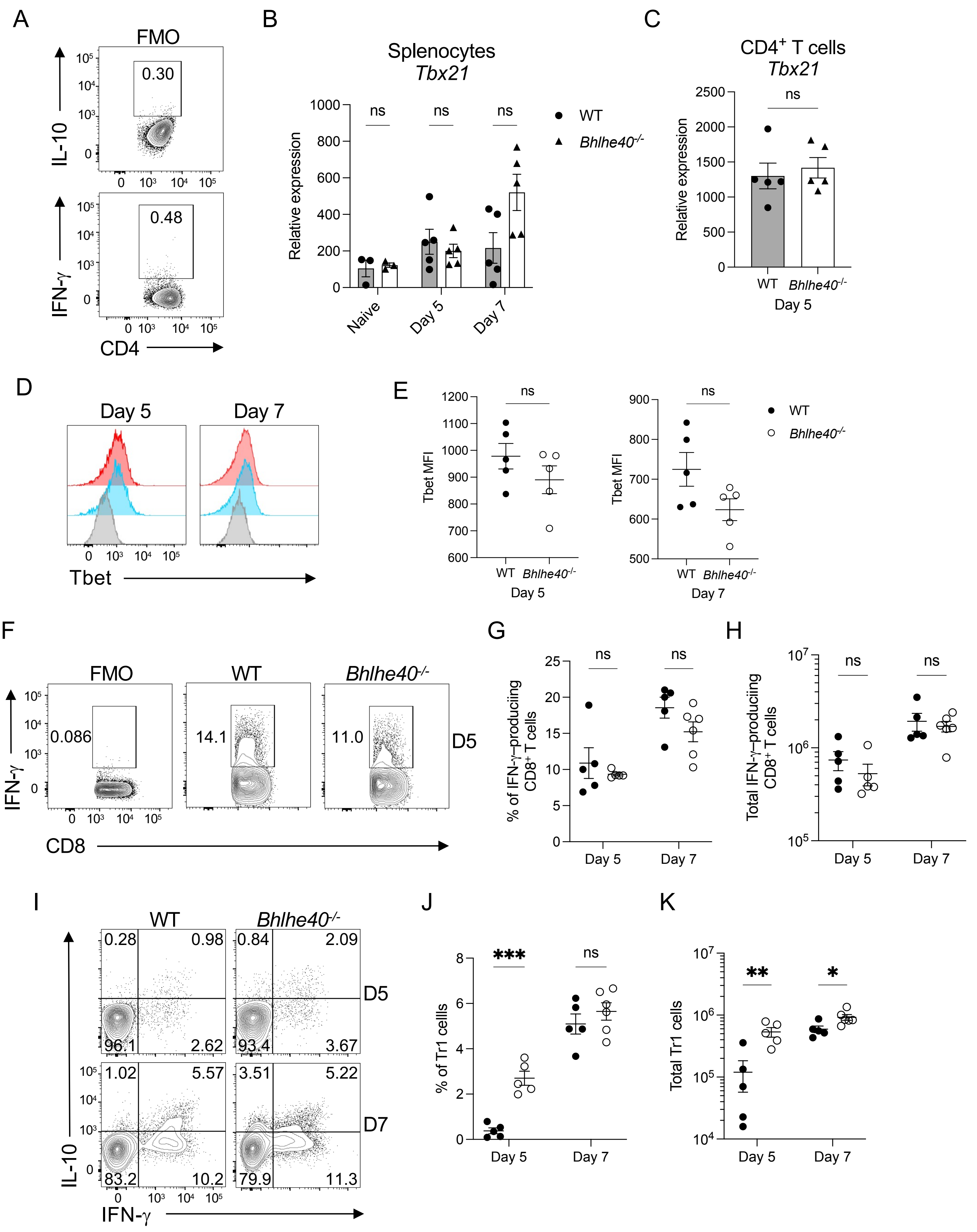
A
FMO
IL-10
IFN-γ
CD4
0.30
0.48
B
Splenocytes
Tbx21
Relative expression
ns
WT
Bhlhe40-/-
Naive
Day 5
Day 7
C
CD4+ T cells
Tbx21
Relative expression
ns
WT
Bhlhe40-/-
Day 5
D
Day 5
Day 7
Tbet
E
Tbet MFI
ns
WT
Bhlhe40-/-
Day 5
Day 7
F
FMO
WT
Bhlhe40-/-
0.086
14.1
11.0
D5
IFN-γ
CD8
G
% of IFN-γ–producing CD8+ T cells
ns
Day 5
Day 7
H
Total IFN-γ–producing CD8+ T cells
ns
Day 5
Day 7
I
WT
Bhlhe40-/-
0.28
0.98
0.84
2.09
96.1
2.62
93.4
3.67
D5
1.02
5.57
3.51
5.22
83.2
10.2
79.9
11.3
D7
IL-10
IFN-γ
J
% of Tr1 cells
***
ns
Day 5
Day 7
K
Total Tr1 cells
**
*
Day 5
Day 7

FIG S1 WT and *Bhlhe40*$^{-/-}$ mice were infected i.p. with 10^5 *P. yoelii* pRBCs. **(A)** FMOs denoting the gating strategy for identifying IFN-γ and IL-10–producing $CD4^+$ T cells after ex vivo restimulation with PMA and ionomycin. Relative expression of *Tbx21* in **(B)** total splenocytes isolated from the spleen of naïve mice and mice infected for 5 and 7 days or **(C)** sort-purified $CD4^+$ T cells isolated from the spleen of mice infected for 5 days as determined by RT-qPCR. Data were normalized to *Hprt*, and the $2^{-\Delta Ct}$ method was used to calculate relative expression. **(D)** Representative histogram plots showing Tbet expression in live, single cell $CD4^+$ T cells from the spleen of WT and *Bhlhe40*$^{-/-}$ mice at days 5 and 7 p.i. (**E**) Median fluorescent intensity for Tbet in WT and *Bhlhe40*$^{-/-}$ $CD4^+$ T cells on days 5 and 7 p.i. (**F**) Representative flow plots of live, single cell IFN-γ^+ $CD8^+$ T cells from the spleen of WT and *Bhlhe40*$^{-/-}$ mice at day 5 p.i. following *ex vivo* restimulation with PMA and ionomycin in the presence of Brefeldin A. Gate based on FMO control. **(G)** Frequency and total number (**H**) of live, single cell IFN-γ^+ $CD8^+$ T cells at days 5 and 7 p.i. Representative flow plots of live, single cell IFN-γ^+IL-10^+ Tr1 cells from the spleen of WT and *Bhlhe40*$^{-/-}$ mice at days 5 and 7 p.i. following *ex vivo* restimulation with PMA and ionomycin in the presence of Brefeldin A. **(J)** Frequency and total number (**K**) of live, single cell IFN-γ^+IL-10^+ Tr1 cells at days 5 and 7 p.i. (**B-E, I-K**) Data are representative of three independent experiments with 3-6 mice per group. (**F-H**) Data are representative of two independent experiments with 5-6 mice per group. (**B, G, H, J, K**) A two-way ANOVA determined significance followed by a post hoc Holm-Sidak multiple comparisons test. (**C, E**) A nonparametric Mann-Whitney *t*-test determined significance. *$p < 0.05$, **$p < 0.01$. ns, denotes not significant.

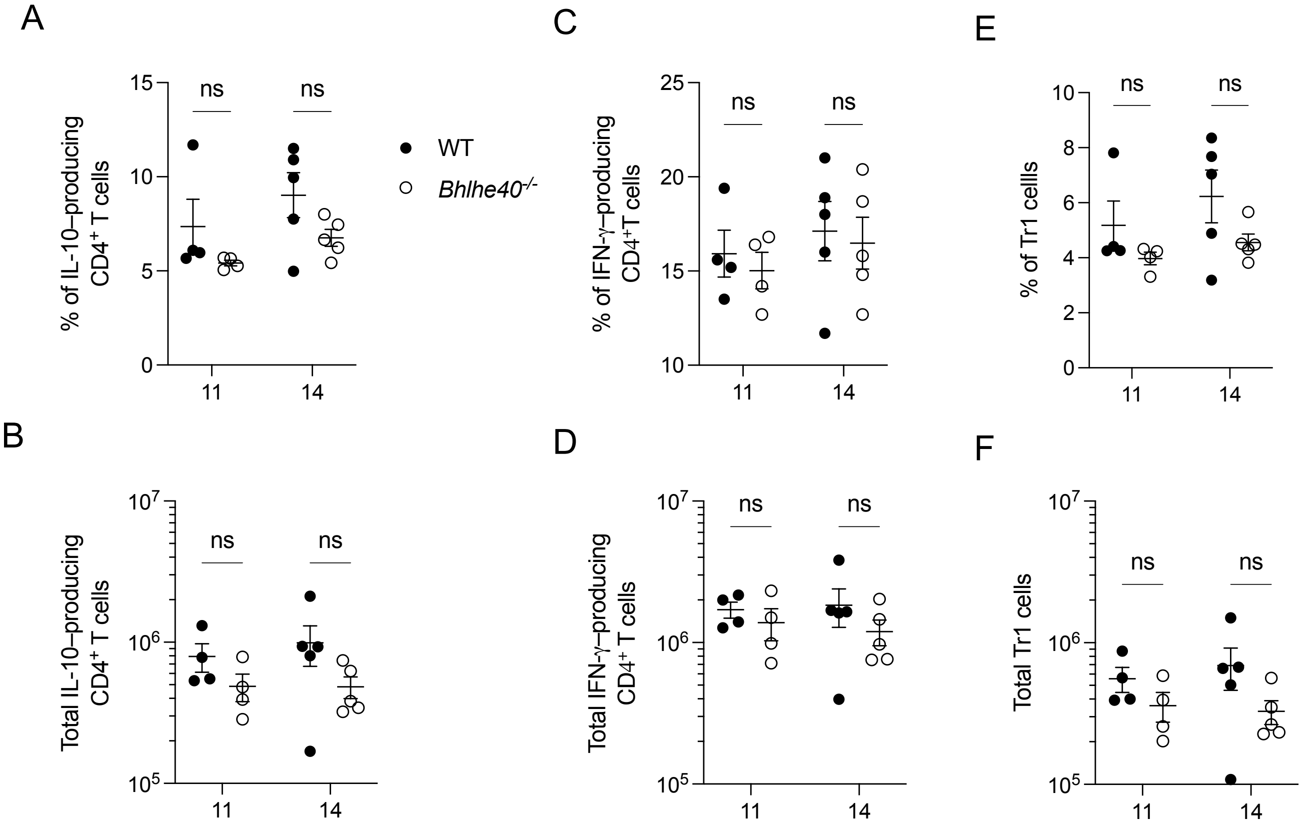

FIG S2 WT and *Bhlhe40*$^{-/-}$ mice were infected i.p. with 10^5 *P. yoelii* pRBCs. Frequency **(A)** and total number **(B)** of live, singlet IL-10–producing CD4$^+$ T cells isolated from the spleen at days 11 and 14 p.i. Frequency **(C)** and total number **(D)** of live, singlet IFN-γ–producing CD4$^+$ T cells isolated from the spleen at days 11 and 14 p.i. Frequency **(E)** and total number **(F)** of live, singlet Tr1 cells isolated from the spleen at days 11 and 14 p.i. Data are representative of two independent experiments with 4-5 mice per group. A two-way ANOVA determined significance followed by a post hoc Holm-Sidak multiple comparisons test. ns, denotes not significant.

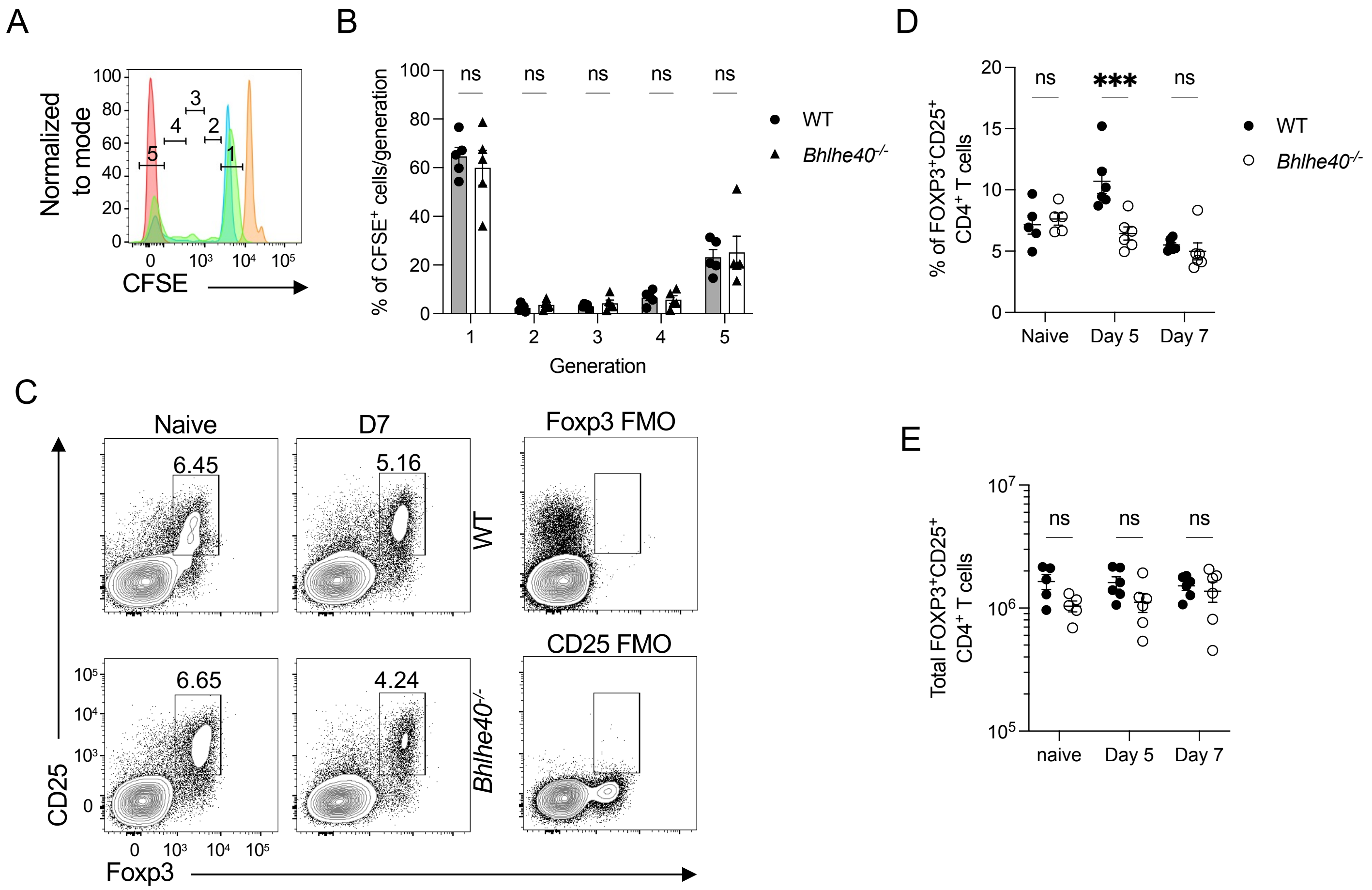

FIG S3 WT and *Bhlhe40*$^{-/-}$ mice were infected i.p. with 10^5 *P. yoelii* pRBCs. (**A**) Representative histogram plot showing CFSE fluorescence of recovered WT (blue) and *Bhlhe40*$^{-/-}$ (green) donor CD4$^+$Thy1.2$^+$ T cells on day 5 after P. yoelii 17X infection. CFSE$^+$ CD4$^+$ T cells prior to transfer (orange) and unlabeled Thy1.1$^+$ CD4$^+$ T cells (red) serve as positive and negative controls for CFE staining. (**B**) Frequency of live, singlet, Thy1.2$^+$ donor CD4$^+$ T cells in each peak highlighted in (A). Donor T cells were recovered five days after infection with P. yoelii 17X and identified based on Thy1.2 staining. (**C**) Representative flow plots of live, singlet, CD25$^+$Foxp3$^+$ CD4$^+$ T cells from the spleen of naïve or day 7 infected WT and *Bhlhe40*$^{-/-}$ mice. Frequency (**D**) and total number (**E**) of live, singlet, CD25$^+$Foxp3$^+$ CD4$^+$ T cells from the spleen of naïve mice and mice infected for 5 and 7 days. Data are representative of two independent experiments with 4-5 mice per group. A two-way ANOVA determined significance followed by a post hoc Holm-Sidak multiple comparisons test. ns, denotes not significant.

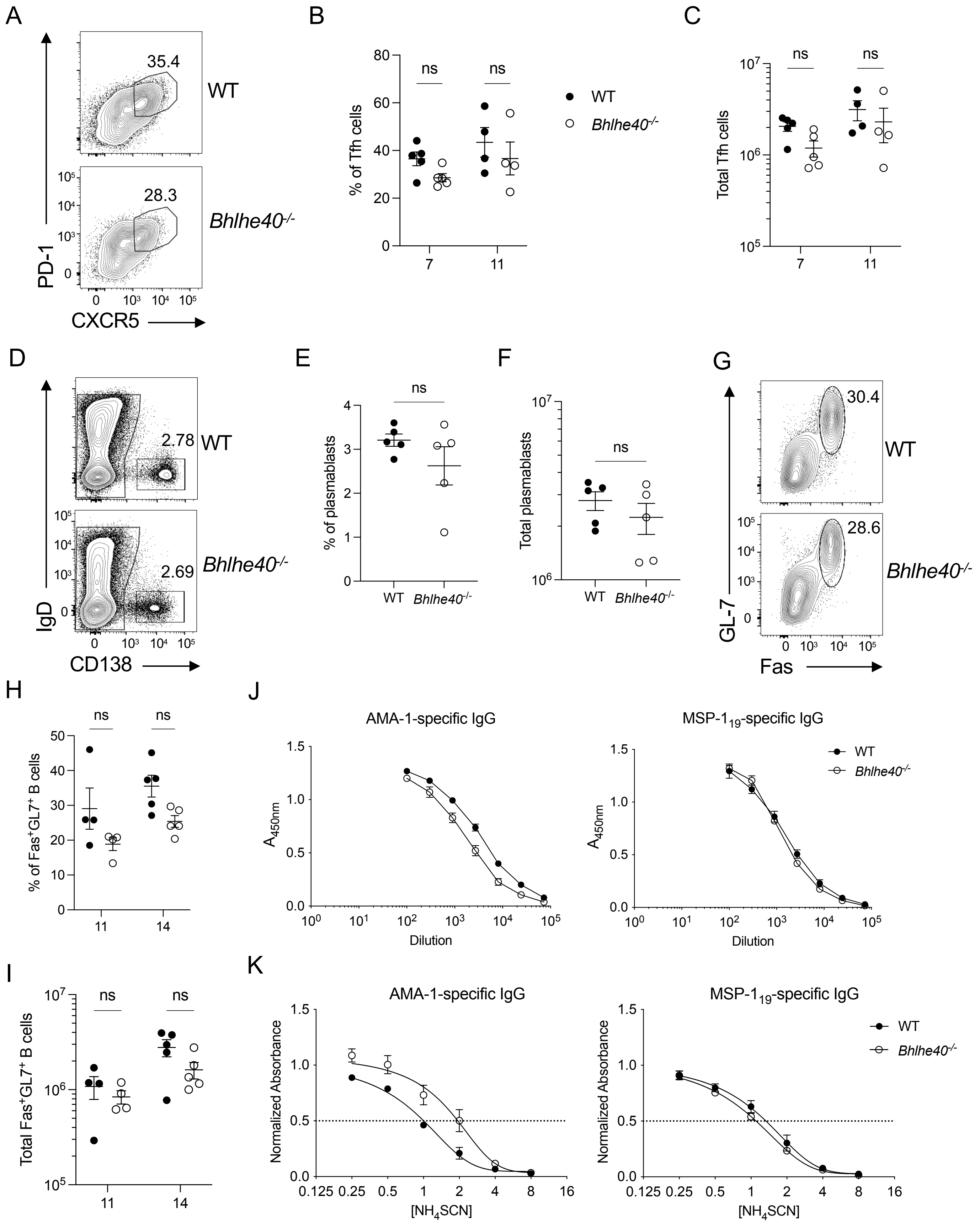

A
35.4
WT
28.3
Bhlhe40-/-
PD-1
CXCR5
B
% of Tfh cells
ns
ns
7
11
WT
Bhlhe40-/-
C
Total Tfh cells
ns
ns
7
11
D
2.78
WT
2.69
Bhlhe40-/-
IgD
CD138
E
% of plasmablasts
ns
WT
Bhlhe40-/-
F
Total plasmablasts
ns
WT
Bhlhe40-/-
G
30.4
WT
28.6
Bhlhe40-/-
GL-7
Fas
H
% of Fas+GL7+ B cells
ns
ns
11
14
I
Total Fas+GL7+ B cells
ns
ns
11
14
J
AMA-1-specific IgG
MSP-1₁₉-specific IgG
A450nm
Dilution
WT
Bhlhe40-/-
K
AMA-1-specific IgG
MSP-1₁₉-specific IgG
Normalized Absorbance
[NH4SCN]
WT
Bhlhe40-/-

FIG S4 WT and *Bhlhe40*$^{-/-}$ mice were infected i.p. with 10^5 *P. yoelii* pRBCs. (**A**) Representative flow plots of live, singlet, CD44^{hi}CD11a$^+$ CXCR5$^+$PD-1$^+$ Tfh cells from the spleen of WT and *Bhlhe40*$^{-/-}$ mice infected for 7 days. Frequency (**B**) and total number (**C**) of live, singlet, CD44^{hi}CD11a$^+$ CXCR5$^+$PD-1$^+$ Tfh cells isolated from the spleen 7 and 11 days p.i. (**D**) Representative flow plots of live, single cell CD138$^+$IgD$^-$ plasmablasts from the spleen 7 days p.i. Frequency (**E**) and total number (**F**) live single cell CD138$^+$IgD$^-$ plasmablasts from day 7 p.i. (**G**) Representative flow plots of live, single cells CD19$^+$B220$^+$ Fas$^+$GL7$^+$ GC B cells from day 11 p.i. Frequency (**H**) and total number (**I**) of live, singlet CD19$^+$ B220$^+$ Fas$^+$GL7$^+$ GC B cells isolated from the spleen of mice infected for 11 and 14 days. (**J**) Serum AMA-1–specific and MSP-1_{19}–specific IgG was determined by ELISA on day 23 p.i. (**K**) Normalized absorbance of AMA-1–specific and MSP-1_{19}–specific IgG on day 23 p.i. as determined by ELISA displayed as a nonlinear regression curve fit. The x-axis (log2) displays increasing molar concentrations of NH_4SCN. The intersection of the dotted line at the normalized absorbance of 0.5 represents the concentration of NH_4SCN needed to elute 50% of the bound IgG off the Ag. Data are representative of two independent experiments with 4-5 mice per group. (**B, C, H-K**) A two-way ANOVA determined significance followed by a post hoc Holm-Sidak multiple comparisons test. (**E, F**) A nonparametric Mann-Whitney *t*-test determined significance. ns, denotes not significant.

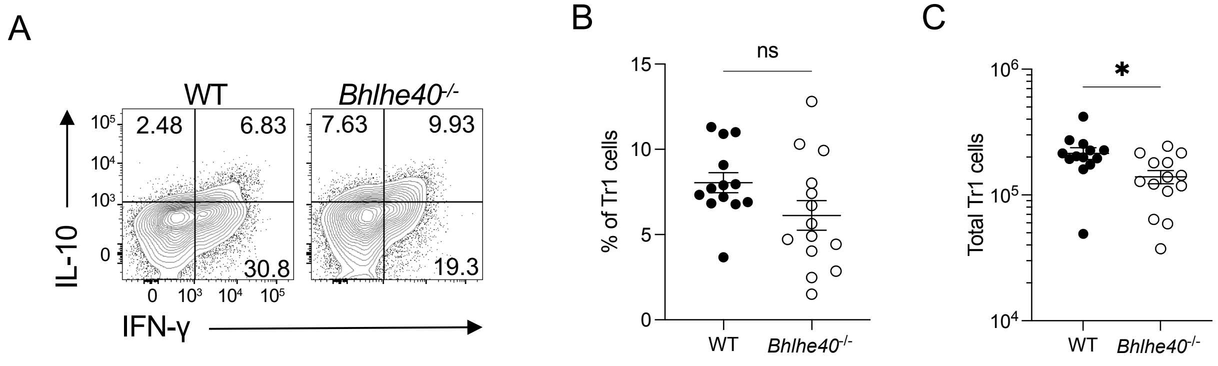

FIG S5 WT and *Bhlhe40*$^{-/-}$ mice were infected i.p. with 10^5 *P. yoelii* pRBCs. (**A**) Representative flow plots of live, singlet IFN-γ^+IL-10^+ Tr1 cells from the liver of WT and *Bhlhe40*$^{-/-}$ mice at day 7 p.i. following *ex vivo* restimulation with PMA and ionomycin in the presence of Brefeldin A. Frequency (**B**) and total number (**C**) of live, singlet, IFN-γ^+IL-10^+ Tr1 cells from the liver at day 7 p.i. Data are pooled from three independent experiments with 3-5 mice per group. A nonparametric Mann-Whitney *t*-test determined significance. *$p < 0.05$, ns, denotes not significant.

TABLE SI List of primer sequences used for quantitative real-time PCR analysis.

Gene	Primer Forward 5’-3’	Primer Reverse 5’-3’
Bhlhe40	ACG GAG ACC TGT CAG GGA TG	GGC AGT TTG TAA GTT TCC TT GC
Il10	CTG GAC AAC ATA CTG CTA ACC	GGG CAT CAC TTC TAC CAG GTA
Ifng	CCC TCA CAC TCA GAT CAT CTT	GCT ACG ACG TGG GCT ACA G
Tbx21	AGC AAG GAC GGC GAA TGT T	GGG TGG ACA TAT AAG CGG TTC
Hprt	GGC TAT AAG TTC TTT GCT GAC CTG	AAC TTT TAT GTC CCC CGT TGA

TABLE SII List of antibodies used for flow cytometry, including the fluorophore, clone, catalog number, and manufacturer.

Antibody	Fluorophore	Clone	Catalog #	Company
CD4	AF700	RM4-5	100536	BioLegend
CD62L	APC	MEL-14	17-0621-83	Invitrogen
GL7	APC	II/41	50-5790-82	Invitrogen
IL-10	APC	JESS-16E3	17-7101-82	Invitrogen
Streptavidin	APC		20-4317-u100	Tonbo
CXCR5	Biotin	L138D7	145510	BioLegend
CD19	BV510	6D5	115546	BioLegend
CD3e	BV510	17A2	100233	Biolegend
CD25	BV605	PC61	102036	BioLegend
CXCR5	BV605	L138D7	145513	BioLegend
B220	BV650	RA3-6B2	103241	BioLegend
CD3	BV711	17A2	100241	BioLegend
CD11a	eF450	M17/4	48-0111-82	Invitrogen
IgD	eF450	11-26c	48-5993-82	Invitrogen
CD45	eF450	30-F11	48-0451-80	eBioscience
Foxp3	eF450	FJK-16s	48-5773-82	Invitrogen
Thy1.2 (CD90.2)	eF450	53-2.1	48-0902-82	Invitrogen
Tbet	eF660	eBio4B10	50-5825-82	Invitrogen
Fixable viability dye	eF780		65-0865-14	Invitrogen
CD11a	FITC	M17/4	101106	BioLegend
IgM	FITC	II/41	11-5790-81	Invitrogen
Ly6C	FITC	HK1.4	128006	BioLegend
TNF	FITC	MP6-XT22	506304	BioLegend
TCRVa2	FITC	B20.1	127820	BioLegend
B220	PercpCy5.5	RA3-6B2	103236	BioLegend
CD3e	PercpCy5.5	145-2C11	45-0031-82	Invitrogen
CD38	PercpCy5.5	90	102722	BioLegend
CD11b	PercpCy5.5	M1/70	65-0112-u100	Tonbo
CD11c	PercpCy5.5	N418	65-0114-u100	Tonbo
Ter119	PercpCy5.5	TER119	65-5921-u100	Tonbo
CD44	PE	IM7	103008	BioLegend
CD45.2	PE	104	50-0454-u100	Tonbo
CD138	PE	281-2	142504	BioLegend
GM-CSF	PE	MP1-22E9	505406	BioLegend
CD11a	PE-Cy7	M17/4	101122	BioLegend
CD62L	PE-Cy7	MEL14	104418	BioLegend
Ki-67	PE-Cy7	SolA15	25-5698-82	Invitrogen
Fas (CD95)	PE-Cy7	Jo2	557653	BD Biosciences
IFN-γ	PE-Cy7	XMG1.2	25-7311-82	Invitrogen
PD-1	PE-Cy7	J43	25-9985-82	Invitrogen
CD44	violetFluor500	IM7	85-0441-u100	Tonbo
